# Modified lipoproteins in periodontitis: a link to cardiovascular disease?

**DOI:** 10.1042/BSR20181665

**Published:** 2019-03-26

**Authors:** Stefan Ljunggren, Torbjörn Bengtsson, Helen Karlsson, Carin Starkhammar Johansson, Eleonor Palm, Fariba Nayeri, Bijar Ghafouri, Julia Davies, Gunnel Svensäter, Johanna Lönn

**Affiliations:** 1 Occupational and Environmental Medicine Center, and Department of Clinical and Experimental Medicine, Linköping University, Linköping, Sweden; 2 Department of Medical Sciences, Örebro University, Örebro, Sweden; 3 Centre for Oral Rehabilitation, Public Dental Health Care, Linköping, Sweden; 4 PEAS Research Institute, Department of Infection Control, Linköping, Sweden; 5 Pain and Rehabilitation Centre, and Department of Medical and Health Sciences, Linköping University, Linköping, Sweden; 6 Department of Oral Biology, Faculty of Odontology, Malmö University, Malmö, Sweden

**Keywords:** lipoproteins, nLC-MS/MS, periodontitis, periodontal microbiota, two-dimensional gel electrophoresis

## Abstract

There is a strong association between periodontal disease and atherosclerotic cardiovascular disorders. A key event in the development of atherosclerosis is accumulation of modified lipoproteins within the arterial wall. We hypothesise that patients with periodontitis have an altered lipoprotein profile towards an atherogenic form. Therefore, the present study aims at identifying modifications of plasma lipoproteins in periodontitis. Lipoproteins from ten female patients with periodontitis and gender- and age-matched healthy controls were isolated by density-gradient ultracentrifugation. Proteins were separated by 2D gel-electrophoresis and identified by map-matching or by nano-LC followed by MS. Apolipoprotein (Apo) A-I (ApoA-I) methionine oxidation, Oxyblot, total antioxidant capacity and a multiplex of 71 inflammation-related plasma proteins were assessed. Reduced levels of apoJ, phospholipid transfer protein, apoF, complement C3, paraoxonase 3 and increased levels of α-1-antichymotrypsin, apoA-II, apoC-III were found in high-density lipoprotein (HDL) from the patients. In low-density lipoprotein (LDL)/very LDL (VLDL), the levels of apoL-1 and platelet-activating factor acetylhydrolase (PAF-AH) as well as apo-B fragments were increased. Methionine oxidation of apoA-I was increased in HDL and showed a relationship with periodontal parameters. α-1 antitrypsin and α-2-HS glycoprotein were oxidised in LDL/VLDL and antioxidant capacity was increased in the patient group. A total of 17 inflammation-related proteins were important for group separation with the highest discriminating proteins identified as IL-21, Fractalkine, IL-17F, IL-7, IL-1RA and IL-2. Patients with periodontitis have an altered plasma lipoprotein profile, defined by altered protein levels as well as post-translational and other structural modifications towards an atherogenic form, which supports a role of modified plasma lipoproteins as central in the link between periodontal and cardiovascular disease (CVD).

## Introduction

Cardiovascular disease (CVD) is the leading cause of morbidity and mortality globally. There is a strong association between atherosclerotic CVD and periodontal disease, where periodontitis is suggested to play a contributory role in CVD [1]. Both diseases are driven by chronic inflammatory responses.

Periodontal diseases are the most common bacterially induced chronic inflammatory diseases in humans, affecting up to 90% of individuals worldwide [2] with over 7% of the global population suffering from severe periodontitis [3]. A dysbiotic bacterial community is the primary etiologic factor. The host–pathogen interactions produce a destructive inflammatory response leading to ulceration of the gingival epithelium and eventually exposing the bacteria and their products to the bloodstream, thereby initiating a systemic inflammation [4]. An increasing body of evidence suggests that reactive oxygen species (ROS) and proteolytic enzymes (bacteria- and host-derived) are major contributors to the chronic inflammatory reactions of periodontitis and atherosclerosis [1]. Elevated concentrations of inflammatory markers of CVD are found in patients with periodontitis [5]. Associations between the two diseases have been found both by comparing different clinical parameters of periodontitis with CVD, or CVD outcome. Data have also provided evidence of a direct relationship between periodontal microbes and subclinical atherosclerosis [6]. Furthermore, a high bacterial burden in periodontal pockets has been shown to be a risk factor for acute coronary syndrome [7]. Both bacterial DNA and live oral bacteria have been found within atherosclerotic plaques and the innate and adaptive immune responses against the bacteria have been proposed to contribute to the development of atherosclerosis [8,9]. Furthermore, experimental animal models demonstrate that infection with periodontal pathogens accelerates atherogenesis [10]. The infection can either play a role in initiating the disease or aggravate an already established atherosclerotic plaque [11,12].

In previous studies we have found that *Porphyromonas gingivalis*, which is a major etiological pathogen of adult periodontal disease [13], induces production of ROS, lipid peroxidation, antioxidant consumption and aggregate formation in an *ex vivo* human whole blood system [14,15]. Our previous findings support a key role for *P. gingivalis* in the link between periodontal and CVD, especially through the potent capacity of the gingipains to proteolytically cleave and modify plasma lipoproteins [15,16].

The development of atherosclerosis is proposed to be initiated by qualitative changes of endothelial cells that line the inner arterial surface. When subjected to an irritating stimulus, such as hypertension or pro-inflammatory mediators (e.g. certain bacterial products, cytokines, high cholesterol levels and modified lipoproteins), the endothelium increases the expression of adhesion molecules that capture leucocytes which then can enter the intima. In parallel, changes in endothelial permeability and composition of the extracellular matrix promote the entry of cholesterol-containing low-density lipoprotein (LDL) particles [17]. Enzymatically or non-enzymatically modified LDL, has been suggested to be involved in the initial phase, as well as during the progression, of atherosclerosis. Monocytes have a crucial role in inducing and maintaining the inflammatory process in the atherosclerotic plaque by recruiting other immune cells, internalising modified LDL and forming foam cells. The retention of modified LDL in subendothelial extracellular matrix and its internalisation by monocytes/macrophages is a hallmark of atherosclerosis [18,19].

High-density lipoprotein (HDL) has anti-atherogenic properties, primarily by reverse cholesterol transport (transport of lipids from peripheral tissues to liver and intestine) and by protecting lipoproteins from oxidation. HDL contains antioxidative enzymes and proteins, such as serum paraoxonase/arylesterase (PON1) and serum paraoxonase/lactonase 3 (PON3), which are involved in the defence against free radical production [20,21]. When pathogenic or environmental stress elevates ROS production (or oxidation via enzymatic reactions) to an extent where the concentrations exceed the protective levels of antioxidants, it may result in oxidation and damage of lipids, proteins and DNA. This is suggested to be an underlying mechanism of atherosclerosis and cancer [22].

The main function of LDL and HDL is to transport lipid molecules to and from different tissues and organs. They also adsorb and transport foreign toxic products, such as bacterial endotoxin, that enters the body [23]. Different subgroups of lipoprotein particles contain diverse apolipoproteins (apo), which play a crucial role in lipoprotein metabolism and are necessary for receptor interactions. The main apolipoprotein in LDL and very LDL (VLDL) is apoB-100, which binds to the LDL-receptor that mediates endocytosis of cholesterol-rich particles. In HDL, the dominating apolipoprotein is apoA-I that is necessary for reverse cholesterol transport. Elevated or decreased levels of lipoproteins are risk factors of CVD [24], and changes in the structure of the lipoprotein particles may alter their function.

Based on our and others’ findings, we hypothesise that patients with periodontitis have a changed plasma lipoprotein profile towards an atherogenic form. Modified lipoproteins may be a key link in the association between periodontal and CVD.

The present study aims at identifying alterations of plasma lipoproteins in patients with periodontitis by analysing the protein expression and oxidation of LDL and HDL particles.

## Materials and methods

### Study participants and protocol

In compliance with the Helsinki Declaration, the present study protocol was approved by the Ethical Committee at Linköping University, Linköping, Sweden (Dnr 2014/119-31). All participants gave written informed consent. The present study has a case–control design. Ten female patients referred between 2015 and 2016 to Centre for Oral Rehabilitation, Linköping, Sweden, a specialist clinic for periodontology, were recruited. Inclusion criteria were generalised severe periodontitis with >30% bone loss at >30% of the teeth and periodontal disease in need of treatment. The control group consisted of ten age-matched females recruited from the department staff, all without any signs of alveolar bone loss. Exclusion criteria were any known chronic inflammatory disease, ongoing infection or malignancy. One of the control subjects was excluded from the study since the clinical chemistry test revealed ongoing inflammation. Therefore, results from nine control subjects are reported.

One periodontal specialist (C.J.S.) performed all examinations. Full-set radiographs were taken. All teeth except the third molars were considered when counting the number of teeth. The periodontal examination included determination of probing pocket depth (PPD), bleeding on probing (BOP), registration of furcation involvement and plaque index (PI). PPDs were recorded on four surfaces (mesial, buccal, distal, lingual) of each tooth according to the manual periodontal probe PCP 11 (Hu-Friedy, Chicago, Illinois, United States). The pocket depths were recorded if they were 4 mm or deeper. BOP was recorded after probing the pockets. The percentage of total number of sites that bled was recorded for each participant.

The periodontal diagnosis was established according to criteria of the American Academy of Periodontology for human periodontitis [25]. The periodontal condition and characteristics of the study subjects are shown in Table 1.

**Table 1:** Demographic and clinical periodontal data of the patients and controls

	Controls (*n*=9)	Patients (*n*=10)
Age mean ± S.D., range	56.9 ± 7.0	57.2 ± 5.7
Number of remaining teeth mean ± S.D.,	27 ± 1.5	25.7 ± 2.7
Range	24–28	21–28
Plaque, % of sites mean ± S.D.,	4.4 ± 4.8	27.3 ± 23.4^2^
Range	0–13	1–62
BOP, % of sites mean ± S.D.,	1.0 ± 1.6	22.5 ± 17.7^2^
Range	0–5	1–52
PPD 4–6 mm % of sites mean ± S.D.,	0.9 ± 0.8	20.4 ± 13.4^2^
Range	0–2	4–39
PPD > 6 mm % of sites mean ± S.D.,	0	2.7 ± 3.8
Range		0–12
Furcation involvement ≥II	0.1 ± 1.4	1.5 ± 1.4^2^
Gingival crevicular fluid (µl)	0.2 ± 0.1	1.0 ± 0.4^2^
Smokers, *n*	0	4
Body Max Index (BMI)	22.7 ± 2.7	27.2 ± 3.2^1^

*t*test for independent samples by group. ^1^*P*≤0.01. ^2^*P*≤0.001.

### Blood sampling

Peripheral venous blood was collected in plasma vacutainer tubes (EDTA, heparin, serum, and glucose tubes (Becton Dickinson [BD] Vacutainer™, Becton Dickinson and Company, Wokingham, U.K.) after at least 4 h of fasting. Samples were taken before periodontal and radiographic examinations and collection of subgingival plaque. The blood was centrifuged at 2200×***g*** for 10 min, and plasma was collected and stored on ice until frozen at −80°C.

### Subgingival plaque sampling and microbiological analysis

Subgingival microbial samples were collected and pooled from four sites; the deepest periodontal pocket in each quadrant or, in controls without deep pockets, from the mesial site of each first premolar. Supra-gingival plaque was removed and the root surface air-dried. A sterile endodontic paper point was inserted into the periodontal pocket for 20 s to collect the bacterial sample, and then transferred to a sterile test tube and stored at −80°C until further analysis. At the Department of Oral Microbiology and Immunology, University of Gothenburg, Sweden, the samples were processed and analysed for the presence of 22 bacterial species associated with subgingival sites and periodontal disease and oral disease/caries and health using a checkerboard DNA–DNA hybridisation technique [26].

### Blood chemistry tests

Blood chemistry tests commonly used for evaluation of health status were performed on plasma at the routine laboratory at the Department of Clinical Chemistry, Linköping University Hospital, Linköping. Analytes measured were: apo-B (LDL), apoA-I (HDL), apoB/apoA-I ratio, C-reactive protein (CRP), blood cell status, creatinine (kidney status), glucose (diabetes), albumin, iron, haemoglobin, transferrin, mean corpuscular haemoglobin (MCH) and MCH concentration (MCHC), alanine aminotransferase (ALAT; liver status) and thyrotropin (thyroid status).

### Lipoprotein isolation

LDL/VLDL and HDL were isolated from EDTA plasma by density-gradient ultracentrifugation as previously described [27]. In short, plasma was mixed with a sucrose and EDTA solution and overlaid with a KBr/PBS solution with density 1.063 g/ml. Centrifugation was performed at 290000×***g*** at 15°C for 4 h in a Beckman Coulter Ti 70.1 rotor (Beckman Instruments, Inc., Palo Alto, CA). The LDL/VLDL and HDL fractions were extracted separately and mixed with KBr/PBS (density 1.24 g/ml) before being subjected to a second round of ultracentrifugation with the same conditions for 2 h. LDL/VLDL and HDL were aspirated from the top of the tubes and desalted using PD-10 desalting columns (GE Healthcare, Little Chalfont, U.K.).

### Two-dimensional gel electrophoresis

Proteins were separated on 2D gels as described previously [28]. Briefly, 400 µg HDL or 300 µg LDL/VLDL proteins were iso-electrically focused on immobilised pH gradient strips (pH 3–10) at 46000 Vh (max 8000 V), then separated overnight on homogenous (T = 14, C = 1.5%) gels. For detection of oxidised proteins, in-gel derivatisation was done using 10 mM 2,4-dinotrophenylhydrazine in 2 M HCl for 20 min followed by 15-min washing in 2 M Tris in 30% glycerol as described previously [23]. The IPG strips were equilibrated in 50 mM Tris/HCl buffer pH 6.8, 35% v/v glycerol, 6 M urea, 2% w/v SDS and 65 mM DTT for 10 min followed by an additional 10 min incubation using a buffer with iodoacetamide (IAA) instead of DTT and a trace of Bromophenol Blue. Samples were transferred to a separate homecast 10% SDS/polyacrylamide gel (SDS/PAGE) with a gradient on GelBond PAG film (T = 11–18%, C = 1.5%) at 30–35 mA overnight and proteins from the gel were further electroblotted. Proteins from the gels were fixed and detected using fluorescent SYPRO Ruby protein stain (Bio-Rad, Stockholm, Sweden) for HDL and silver stain for LDL. Gel images were evaluated using PDQuest 2-D gel analysis software, version 7.1.0 (Bio-Rad). Protein abundance was expressed as percent of total 2D gel fluorescence. Multiple isoforms of proteins were both analysed separately but also added together. Protein spots detected on 2D gels were map-matched [27,29].

### OxyBlot

SDS/PAGE gels were blotted on Immun-Blot PVDF membrane using Trans-Blot Electrophoretic Transfer Cell (Bio-Rad). Membranes were blocked in TBS (40 mM Tris/HCl, 500 mM NaCl, pH 7.5) with 5% non-fat dried milk overnight. Membranes were washed with Tween-20 TBS (TTBS: 40 mM Tris/HCl, 500 mM NaCl, 0.05% Tween-20) and incubated with primary antibody directed to carbonylated proteins with 1:7500 anti-DNPH antibodies (Sigma–Aldrich, St. Louis, MI, U.S.A.) in TTBS with 2% non-fat dried milk overnight. The membranes were washed with TTBS followed by incubation with horseradish peroxidase–conjugated secondary antibody (antigoat/sheep IgG, Sigma–Aldrich) for 1 h. The latter wash procedure was repeated once followed by detection of antigen/antibody conjugate with the ECL Prime Western blotting detection system (GE Healthcare) and developed on X-ray film.

### Nanoliquid tandem MS

Proteins were analysed with nanoliquid tandem MS (nLC-MS/MS) similar to a previously described method [30]. In short, desalted lipoproteins were lyophilised and reconstituted in 8 M urea in 25 mM ammonium bicarbonate. Proteins were reduced with DTT and alkylated with IAA followed by a buffer change to 25 mM ammonium bicarbonate using an Amicon 3 kDa cutoff filter (Millipore, Molsheim, France). Protein concentration was measured using 2-D Quant Kit (GE Healthcare) and 10 µg of proteins were digested with trypsin (1:25, Promega, Madison, WI, U.S.A.) for 1 h in a sonication bath. The peptides were dried with a vacuum centrifugation system and reconstituted in 0.1% formic acid in water.

Reconstituted peptides corresponding to 250 ng of proteins were loaded into a nano liquid chromatography system (EASY-nLC, Thermo Scientific, Waltham, MA, U.S.A.) with a C18 column (100 mm × 0.75 µm, Agilent Technologies, Santa Clara, CA, U.S.A.). Peptides were separated using a 90-min increase from 4 to 40% acetonitrile, supplemented with 0.1% formic acid, followed by an increase to 90% acetonitrile for 10 min. Peptides were analysed by a data-dependent acquisition method utilising collision-induced dissociation for sequencing on an LTQ Velos Orbitrap Pro mass spectrometer (Thermo Scientific).

MS data were analysed with MaxQuant v1.5.3.12 (Max Planck Institute of Biochemistry, Martinsried, Germany) utilising the human Uniprot/Swissprot database (downloaded on 3 March 2017). A mass tolerance of 5 ppm for MS search and 0.5 Da for MS/MS search, as well as a false-discovery rate of 1%, were used. Proteins with at least one unique peptide and found in >50% of the samples were considered identified and used in subsequent analyses. Protein quantities were expressed as LFQ (label-free quantification) intensity from the software.

### Methionine oxidation of apoA-I in HDL and LDL/VLDL

Methionine oxidations of apoA-I in HDL and LDL/VLDL were investigated using data obtained by nLC-MS/MS. Peptide intensities of methionine residues at positions 110, 136 and 172 (including pre- and propeptide), previously found to be oxidised [28], were analysed in the mass spectrum data.

### Determination of oxidised LDL, total antioxidant capacity and NO metabolites

Human oxidised LDL in serum was determined by OxiSelect™ Human Oxidized LDL ELISA kit (OxPL-LDL Quantification, Cell Biolabs, San Diego, CA, U.S.A.) in serum samples collected from patients and controls as described above. The method quantifies the level of oxidised phospholipids associated with human LDL, and the measurement was performed according to the manufacturer’s recommendations. Total antioxidant capacity (PAO test kit; KPA-050, JaICA, Shizuoka, Japan), and the total nitric oxide metabolites; nitrite and nitrate (KGE001; R&D systems, Minneapolis, MN) were measured in serum according to the manufacturers’ recommendations. Absorbance was measured at 450 nm for oxidised LDL and 480 nm for total antioxidants by using a Multiskan Ascent microplate reader (Thermo Labsystem, Stockholm, Sweden), and NO metabolites at 540 nm with correction at 690 nm by using Spectramax 190 plate reader (Molecular Devices, Sunnyvale, CA).

### PON1 activity in serum

The levels of the antioxidant PON1 arylesterase activity in serum samples were determined in a similar way to a previously described method [28]. Serum was diluted 1:80 in salt buffer (20 mM Tris/HCl, 1.0 mM CaCl_2_, pH 8.0) and a triplicate of 20 μl diluted serum were added to the wells in a UV-transparent 96-well plate (Sigma–Aldrich,). A total of 200 μl of phenyl acetate solution, containing 3.26 mM phenyl acetate in salt buffer, were added to each well and the absorbance of produced phenol was measured at 270 nm in a Clariostar plate reader (BMG Labtechnologies, Offenburg, Germany). The initial period when the reaction was linear were used for calculation of activity, expressed as U/ml, using an extinction coefficient of phenol of 1310 M^−1^.cm.

### Serum amyloid A1 ELISA

To investigate the acute phase response by serum amyloid A (SAA), EDTA-plasma SAA1 levels were measured using an ELISA (DY3019-05; R&D systems) according to the manufacturers’ instructions. Absorbance was measured at 450 nm with correction at 570 nm using a Spectramax 190 plate reader (Molecular Devices).

### Multiplex immunoassay analysis

The concentrations of 71 cytokines, chemokines and growth factors in EDTA-plasma were analysed in duplicate using a U-PLEX assay based on an electrochemiluminescent detection method (Meso Scale Diagnostics, Rockville, MD, U.S.A.) according to the manufacturer’s recommendations. Data were collected and analysed using MESO QUICKPLEX SQ 120 instrument equipped with DISCOVERY WORKBENCH® data analysis software (Meso Scale Diagnostics, Rockville, MD, U.S.A.). The precision based on both intra and inter-assays variations were <10% within the detection limits provided by the manufacturer.

### Statistical analysis

The clinical periodontal data were analysed by Student’s *t* test for independent samples, presented as mean ± S.D. The quantification data were not normally distributed and therefore analysed by Mann–Whitney U test. Normally distributed data in the clinical chemistry tests and Multiplex analysis were analysed by Student’s *t*-test, and not normally distributed data by Mann–Whitney U test. Correlation between parameters was analysed by Spearman’s Rank test. Software used were SPSS (SPSS Inc, Armonk, NY, U.S.A.) as well as GraphPad Prism (GraphPad software, La Jolla, CA, U.S.A.). Values were expressed as median (min-max) and *P*≤0.05 was considered as statistically significant. Significance is denoted as: *P<0.05, **P<0.01 and ***P<0.001. In addition, multivariate modeling with orthogonal partial least square discriminate analysis (OPLS-DA) was performed with SIMCA v14.0 (MKS data analytics solutions, Malmö, Sweden). Variables analysed include demographic and clinical periodontal data, microbial species frequencies, blood chemistry tests, cytokines/chemokines as well as proteins obtained by 2D gel electrophoresis (2-DE) and nLC-MS/MS in LDL/VLDL and HDL. To understand which variables were important for separation of the two groups, the variable importance in projection (VIP) value was used.

## Results

The characteristics and periodontal conditions of the patients and controls are shown in Table 1.

### Microbial species in subgingival plaque

**Figure 1:**
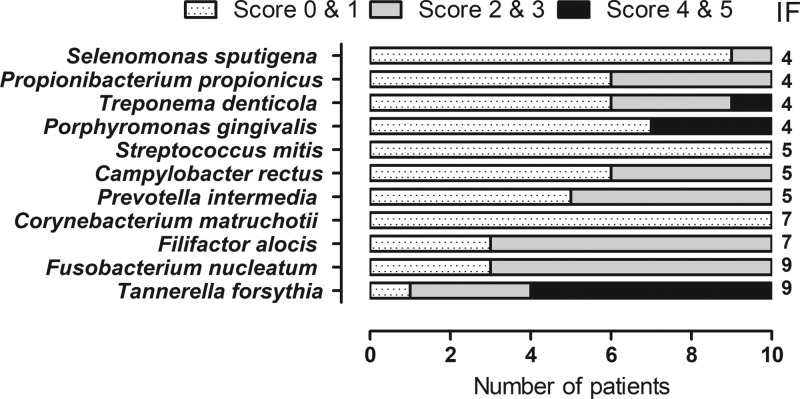
Frequency distribution and amount of bacterial species in the subgingival plaque of ten patients with severe periodontitis investigated using DNA–DNA hybridisation technique The presence of 11 bacterial species associated with subgingival bacterial flora were detected. The number of bacteria found is expressed in a scoring system (0–5), where score 0–1 corresponds to 0–10^5^, score 2–3 corresponds to 10^5–^10^6^ and 4–5 corresponds to ≥10^6^ bacteria. The IF indicate the number of patients in which the species were found.

Of the 22 microbial species tested, 11 were detected in the patient group (Figure 1) and none in the control group. The amount is expressed in a scoring system (0–5), where score 0–1 corresponds to 0–10^5^, score 2–3 corresponds to 10^5^–10^6^ and 4–5 corresponds to ≥10^6^ bacteria. The median number of total bacteria detected in one subject was 161500 (29500–721000, min–max). Species associated with periodontitis were isolated more frequently and in higher amounts. The isolation frequency (IF) of *Tanerella forsythia* and *Fusobacterium nucleatum* was nine out of ten patients. *Filifactor alocis* was found in seven patients, from whom *T. forsythia* and *F. nucleatum* were also isolated. *P. gingivalis* and *Treponema denticola* were found in four patients, all of whom also had *T. forsythia*. In one patient ‘the red complex’, *P. gingivalis, T. forsythia* and *T. denticola*, a group of bacteria that are associated with severe forms of periodontal disease, was found. We did not detect: *Abiotrophia defecta, Actinomyces oris, Aggregatibacter actinomycetemcomitans, Capnocytophaga ochracea, Gemella morbillorum, Granulicatella adiacens, Haemophilus parainfluenzae, Lactobacillus salivarius, Neisseria mucosa, Parvimonas micra* or *Veillonella parvula* in the subgingival sites of either the patients or controls.

### Blood chemistry tests

The levels of LDL (apo-B) were increased and HDL (apoA-I) levels were decreased, giving an increased apoB/apoA-I-ratio, in the patient group compared with the control group. The levels of glucose, MCH (average amount of haemoglobin in the red blood cells) and MCHC (average amount of haemoglobin in each red blood cell) were also increased (Table 2).

**Table 2:** Clinical chemistry test results of plasma from patients with periodontitis and healthy controls

	Control **median**, (min–max)	Patients **median**, (min–max)		Controls **median**, (min–max)	Patients **median**, (min–max)
**ApoA-I (g/l)**	1.8 (1.46–2.15)	1.54^1^ (1.30–2.23)	**Leucocytes (×10^9^/l)**	5.6 (4.0–7.7)	7.1 (4.2–8.0)
**ApoB (g/l)**	0.79 (0.65–1.22)	1.13^1^ (0.89–1.44)	**Platelets (×10^9^/l)**	244 (197–420)	269.5 (204–312)
**ApoB/ApoA-I ratio**	0.45 (0.30–0.82)	0.79^1^ (0.40–0.94)	**Erythrocytes (×10^12^/l)**	4.8 (4.1–5.4)	4.65 (4.3–4.8)
**Glucose (mmol/l)**	5.1 (4.0–5.6)	5.4^1^ (5.0–5.9)	**EVF**	0.47 (0.38–0.48)	0.44 (0.41–0.46)
**CRP (mg/l)**	5 (5–5)	5 (5–6)	**MCV (fl)**	95 (88–102)	95 (90–96)
**ALAT (µkat/l)**	0.45 (0.32–0.76)	0.36 (0.22–0.71)	**MCH (pg)**	28 (27–32)	32^2^ (30–32)
**Thyrotropin (mIE/l)**	1.5 (0.52–2.10)	0.86 (0.58–1.60)	**MCHC (g/l)**	307 (298–312)	334^3^ (323–339)
**Albumin (g/l)**	41.5 (37–47)	45 (39–48)	**Haemoglobin (g/l)**	140 (115–150)	143.5 (138–152)
**Creatinine (µmol/l)**	72 (57–92)	68 (50–87)	**Iron (µmol/l)**	17 (5.5–18.0)	19 (13–23)
**eGFR (ml/min/1.73m^2^)**	72 (56–90)	79 (58–90)	**Transferrin (g/l)**	2.46 (2.2–3.2)	2.55 (1.90–3.15)
			**Transferrin saturation**	0.265 (0.08–0.32)	0.31 (0.17–0.42)

Mann–Whitney U-test or Student’s *t* test. Abbreviations: eGFR, estimation of glomerular filtration rate; EVF, erythrocyte volume fraction. ^1^*P*≤0.05. ^2^*P*≤0.01. ^3^*P*≤0.001.

**Table 3:** Proteins in LDL/VLDL and HDL differing significantly in their expression between patients with periodontitis and controls identified by 2-DE

Protein	Spot number	Uniprot ID	Controls, median (min–max)	Patients, median (min–max)
**LDL/VLDL**				
ApoA-I isoform d	2110	P02647	3886.2 (2458.9–6311.4)	1494.1 (60.1–6087.3)^1^
ApoA-I isoform e	2109	P02647	8249.3 (6425.7–12065.9)	4532.7 (1102.8–11385.6)^2^
**HDL**				
ApoA-I glycated isoform b	2905	P02647	2840.3 (1025.2–4417.6)	1503.55 (243.6–2486.4)^1^
ApoJ isoform a	2913	P10909	727.5 (55.6–1089.1)	391.3 (106.6–595.3)^1^
ApoJ isoform b	2914	P10909	580.8 (437.6–793)	364.45 (12.9–539.2)^3^
ApoJ Total		P10909	1683.3 (517.1–2851.5)	1027.7 (307.5–1449.8)^2^
SAA4 isoform a	9903	P35542	4545.4 (2798.6–6297.6)	2625.05 (1542–4267.7)^2^
SAA4 isoform f	9902	P35542	20955.4 (9645.1–31165.9)	32117.75 (21287–46488.1)^2^

Mann–Whitney U-test. ^1^*P*<0.05. ^2^*P*<0.01. ^3^*P*<0.001.

The differences in the MCH and MCHC between the groups were found to depend on values below the reference interval in the control group. ApoA-I correlated to plasma thyrotropin (r0.79**) and negatively to BMI (r−0.86**), whereas the apoB/apoA-I ratio correlated positively (r0.75**).

### Two-dimensional gel electrophoresis

**Figure 2:**
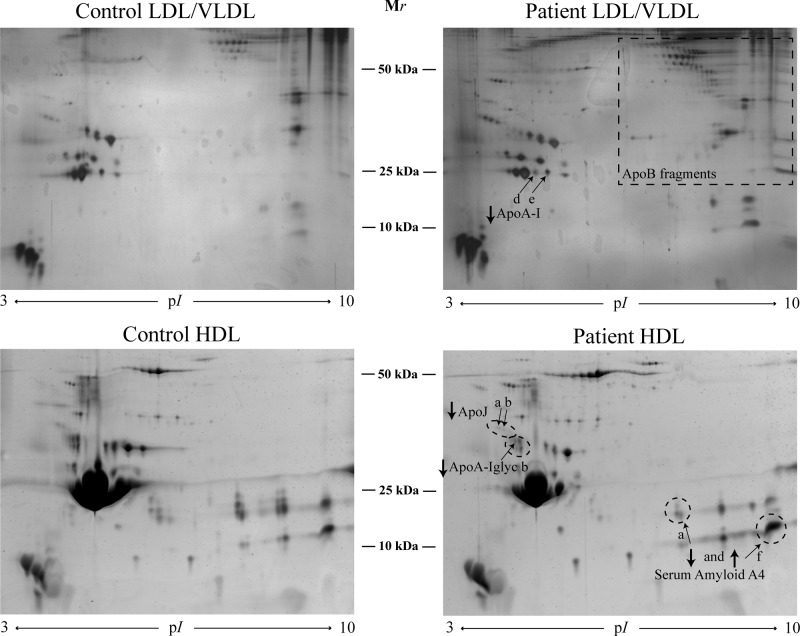
2-DE pattern of LDL/VLDL and HDL proteins from controls and patients A total of 400 and 300 µg of proteins were loaded on HDL and LDL/VLDL gels, respectively. Proteins were visualised by SYPRO Ruby protein staining for HDL and silver staining for LDL/VLDL.

The patients showed decreased levels of apoA-I and its proform compared with the controls (Figure 2, Table 3). There was a positive correlation of the expression of the apoA-I and its proform in LDL to number of teeth (r0.61* and r0.53*, respectively). There was a negative correlation between one apoA-I isoform (e) and one SAA1 isoform (b) (r−0.56). Total apoA-I correlated to total SAA4 (r−0.8**).

In HDL, a glycated isoform of apoA-I, as well as total and two isoforms (a and b, the two most acidic isoforms with lowest pI) of apoJ, were decreased in the patient group (Figure 2, Table 3). There was a negative correlation between the glycated apoA-I in HDL to pocket depth (r−0.58**), especially to the number of deep pocket depths (>6 mm, r−0.68**), and to BOP (−0.48*). All apoJ forms correlated negatively to pocket depths (r between −0.5* and −0.7**). There was a negative correlation between the total apoA1 and SAA1b (right SAA isoform on the 2-DE gel) (r = −0.49*). All protein quantities as found by 2-DE are shown in Supplemental Table 1.

### nLC-MS/MS

In LDL/VLDL, the patients showed decreased levels of apoA-I and increased levels of apoL-1, Ig γ-1 chain C region and platelet-activating factor acetylhydrolase (PAF-AH) compared with the controls (Table 4). The expression of apoM and PON1 was elevated, but the differences were not statistically significant (*P*=0.055 and 0.066, respectively). All protein quantities obtained by nLC-MS/MS are presented in Supplemental Table 2.

**Table 4:** Proteins differing significantly between patients with periodontitis and controls found by nLC-MS/MS

Protein	Gene name	Uniprot ID	Number of identified peptides	Sequence coverage (%)	Controls, median (min–max)	Patients, median (min–max)
**LDL/VLDL**						
**ApoA-I**	APOA1	P02647	43	83.1	2.0E+9 (9.4E+8–2.8E+9)	9.1E+8 (1.4E+8–1.8E+9)^2^
**ApoL1**	APOL1	O14791	21	63.6	1.2E+7 (8.0E+6–2.4E+7)	3.7E+7 (3.7E+6–1.5E+8)^1^
**Ig γ-1 chain C region**	IGHG1	P01857	3	17.3	6.2E+5 (4.0E+5–8.2E+5)	8.3E+5 (4.1E+5–1.3E+6)^1^
**Platelet-activating factor acetylhydrolase**	PLA2G7	Q13093	12	32	3.3E+6 (2.4E+6–5.3E+6)	5.7E+6 (2.5E+6–7.5E+6)^1^
**HDL**						
α**-1-antichymotrypsin**	SERPINA3	P01011	3	11.3	5.9E+5 (3.6E+5–1.3E+6)	1.1E+6 (4.8E+5–1.4E+6)^1^
**ApoA-II**	APOA2	P02652	13	77	1.9E+9 (1.6E+9–2.5E+9)	2.7E+9 (2.1E+9–3.4E+9)^2^
**ApoC-III**	APOC3	P02656	7	55.6	5.0E+8 (4.6E+8–6.0E+8)	7.0E+8 (4.8E+8–1.4E+9)^1^
**ApoF**	APOF	Q13790	6	33.4	2.8E+7 (1.7E+7–5.5E+7)	1.7E+7 (1.1E+7–4.2E+7)^1^
**ApoJ**	CLU	P10909	8	24.5	5.2E+6 (3.9E+6–7.5E+6)	2.8E+6 (1.8E+6–6.9E+6)^1^
**Complement C3**	C3	P01024	23	20.5	2.7E+6 (5.3E+5–2.3E+7)	1.0E+6 (8.7E+5–2.9E+6)^1^
**Ig κ chain C region**	IGKC	P01834	4	67	2.1E+6 (1.6E+6–3.9E+6)	1.7E+6 (3.2E+5–3.1E+6)^1^
**Phospholipid transfer protein**	PLTP	P55058	9	21.9	3.5E+6 (2.3E+6–4.5E+6)	1.8E+6 (7.8E+5–4.0E+6)^1^
**Serum PON3**	PON3	Q15166	8	29.9	2.9E+6 (2.4E+6–5.1E+6)	1.6E+6 (1.5E+5–5.3E+6)^1^

Mann–Whitney U-test. Values are LFQ-intensity obtained through the software. ^1^*P*<0.05.

^2^*P*<0.01.

ApoA-I in LDL/VLDL correlated negatively to SAA1 (r−0.84**) and BOP (r−0.65**), whereas ApoL-1 correlated to BOP (r0.59**). PAF-AH correlated to the number of pocket depths between 2 and 4 mm (r0.5*) and the volume of gingival crevicular fluid (GCF) (r0.6**).

In HDL, the patients expressed decreased levels of apoF, apoJ, complement C3, Ig κ chain C region, PLTP and PON3 and increased levels of α-1-antichymotrypsin, apoA-II, apoC-III compared with controls (Table 4). Phosphatidylcholine-sterol acyltransferase (LCAT) was down-regulated in the patients, although not statistically significant (*P*=0.07). LCAT levels correlated negatively to pocket depths (r−0.5*) and BOP% (r−0.53*).

ApoA-II correlated to pocket depth (r0.67**), BOP (0.7**), GCF volume (r0.75**), and ApoC-III correlated to pocket depth (r0.51*), BOP (r0.63**), GCF volume (r0.75**). ApoJ correlated to BOP (r−0.58**) and ApoF correlated to the number of teeth (r0.53*). Ig κ chain C region correlated negatively to BOP (r−0.6**), pocket depths (r−0.5* for 2–4 mm and r–0.72** for 6 mm). PON3 correlated negatively to BOP (r–0.54*).

### OxyBlot

**Figure 3:**
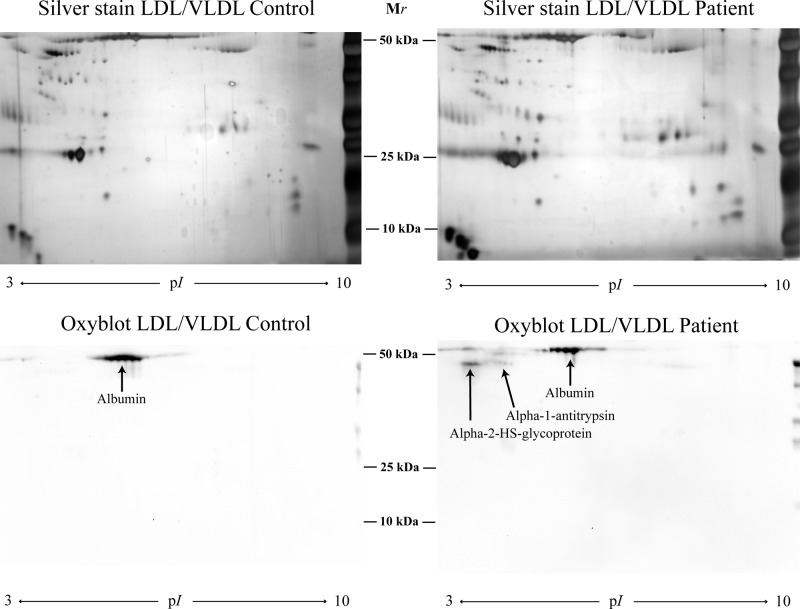
The carbonylation pattern was evaluated by Oxyblot of two 2D gels of LDL/VLDL isolated from one patient with periodontitis and one control

α-1-antitrypsin and α-2-HS-glycoprotein were found to be oxidised in the patient group, and albumin was oxidised in both patients and controls (Figure 3).

### Methionine oxidation

The oxidation of the methionine at positions 110, 136 and 172 in apoA-I in LDL was significantly decreased in the patient group compared with the controls. In HDL there was a significant increase in the methionine oxidation at positions 110 and 136 (Table 5), which correlated to pocket depth (r0.55* and r0.62**, respectively), BOP (r0.6** and r0.59, respectively) and volume of GCF (r0.54* and r0.64**, respectively).

**Table 5:** Apolipoprotein A-I methionine oxidations in patients with periodontitis and controls analysed by nLC-MS/MS

Methionine position	Controls		Patients	
	LDL/VLDL	HDL	LDL/VLDL	HDL
**110**	2.4e+6 (1.1e+6–4.2e+6)	9.9e+6 (5.0e+6–1.5e+7)	1.2e+6 (0.0–1.6e+6)^1^	1.7e+7 (9.4e+6–2.69e+007)^1^
**136**	2.9e+6 (1.2e+6–9.2e+6)	2.9e+6 (1.8e+6–4.3e+6)	9.3e+5 (0.0–2.1e+6)^1^	4.1e+6 (3.0e+6–5.29e+006)^1^
**172**	2.7e+7 (5.5e+6–5.8e+7)	2.0e+8 (1.7e+8–2.4e+8)	6.6e+6 (0.0–2.5e+7)^1^	2.2e+8 (6.8e+7–3.0e+8)

Mann–Whitney U-test.

^1^*P*<0.01.

### OxPL-LDL quantification, total antioxidant capacity and NO metabolites

No significant difference was found in OxPL-LDL between patients and controls (median (min–max); 262.9 (175.3–677.9) and 237.4 (192.7–583.7) µg/ml, respectively), nor in nitrate levels 14.1 (9.3–15.19) and 22.4 (11.4–31.1) µmol/l, respectively. Nitrite levels were below the minimum detection limit. Total antioxidant capacity was higher in the patient group compared with controls (*P*=0.004, 1108 (886.6–1520.0) and 860.5 (646.5–1088) µmol/l, respectively), and correlated to BOP (r0.62**) and volume of GCF (r0.56*).

### Paraoxonase-1 arylesterase activity

There was no difference in serum PON1 arylesterase activity between the patients and controls (median (min–max); 88.5 (69–127) and 93.5 (56–111) U/ml, respectively).

### SAA1

There was no significant difference in plasma SAA1 levels between the patients and controls (median (min–max); 4.5 (2.3–7.5) and 3.4 (1.0–10.8) µg/ml, respectively). The results correlated with both 2-DE (r0.69***) and the nLC-MS/MS data (r0.69***) of SAA1 indicating that the ELISA kit was specific for SAA1.

### Levels of cytokines and chemokines

To investigate the multivariate correlations between the cytokines/chemokines and group membership OPLS-DA was used. Principal component analysis (PCA) was used prior to the OPLS-DA analysis. PCA is an unsupervised analysis that can be used to extract and display systematic variation in the data matrix. The PCA score plots in combination with Hotelling’s T2 (identifies strong outliers) and distance to model in X-space (identifies moderate outliers) showed no outlier. The OPLS-DA model demonstrated clear separation between the groups, but the model had too low R^2^ and Q^2^ values to be acceptable for analysis therefore new OPLS-DA was generated. A total of 17 cytokines/chemokines had a VIP > 1 and were considered important for the group separation (Table 6). Those proteins together explained 46% (R^2^) of the variation and with a prediction of 63% (Q^2^). The CV-ANOVA revealed that the model was significant (*P*=0.034). Among the highest discriminating proteins were IL-21, Fractalkine, IL-17F, IL-7, IL-1RA and IL-2. These differences in concentration (except for IL-1RA) were furthermore found to be significant according to the univariate analysis, indicating that these proteins constitute some of the key differences seen between the groups.

The plasma concentrations of all the 71 inflammatory proteins analysed are presented in Supplemental Table 4.

**Table 6:** Cytokines/chemokines with VIP value > 1 that were significant for group separation (patients compared with controls)

Protein name	Controls (*n*=9) median (min–max) pg/ml	Patients (*n*=10) median (min–max) pg/ml	VIP	Patient compared with Control
**IL-21**	10.0 (4.3–17.0)	18.0 (6.5–38.6)	1.38173	↑^1^
**Fractalkine**	10553.0 (7150–11889)	9137.0 (6695.0–11035.0)	1.27686	↓^1^
**IL-17F**	196.2 (74.0–329.6)	137.3 (37.0–202.4)	1.25763	↓^1^
**IL-7**	1.7 (1.2–2.8)	1.1 (0.8–2.9)	1.23509	↓^1^
**IL-1RA**	174.2 (105.1–340.0)	258.2 (129.9–777.0)	1.21305	↑
**IL-2**	0.5 (0.3–1.2)	0.4 (0.1–0.7)	1.20296	↓
**IL-29**	6.0 (3.5–13.3)	10.3 (3.1–35.8)	1.17863	↑
**GRO-α**	161.9 (24.7–531.8)	50.3 (33.3–292.2)	1.17843	↓
**IL-18**	18.8 (10.9-21.5)	17.7 (12.0–73.2)	1.12634	↓
**MDC**	727.5 (511.0–936.2)	882.6 (509.1–1541.0)	1.10045	↑
**EPO**	88.7 (59.6–145)	72.1 (36.9–103.2)	1.05593	↓
**IL-8**	6.3 (4.1–23.1)	5.1 (3.4–6.7)	1.05578	↓
**IL-17C**	9.7 (0.0–18.7)	5.6 (1.6–17.4)	1.0419	↓
**IL-4**	0.1 (0.0–0.1)	0.0 (0.0–0.1)	1.0211	↓
**IL-23**	7.0 (3.3–10.7)	8.0 (4.0–14.9)	1.01299	↑
**IL-17D**	49.6 (16.8–89.0)	36.4 (26.9–82.1)	1.00649	↓
**IL-2Rα**	557.7 (279.0–718.3)	385.4 (258.4–833.4)	1.00128	↓

Mann–Whitney U-test or Student’s *t*test. ↑ and ↓ indicate up-regulated and down-regulated proteins in plasma from patients with periodontitis compared with controls.

^1^*P*<0.05, indicates significant (*P*<0.05) proteins according to univariate statistic.

### Multivariate modelling

**Figure 4:**
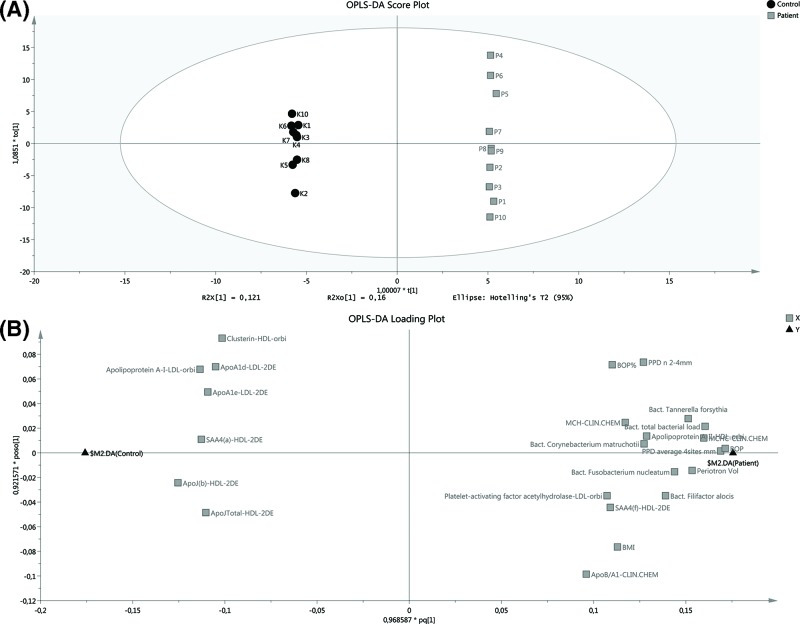
Multivariate modelling of all variables (**A**) Score scatter plot showing the separation of patients and controls along the predictive x-axis. (**B**) Loading plot of variables with a VIP-value >1.5. Variables to the left are decreased while variables to the right are increased in patients compared with controls.

To identify which variables were important for separation of patients and controls, an OPLS-DA regression model for the 271 variables was generated. The model included one predictive and three orthogonal latent variables and showed an excellent fit (R^2^ = 0.99), as well as a good prediction (Q^2^ = 0.84). The CV-ANOVA revealed that the model was highly significant (P<0.005). As can be seen in the score plot, the two groups were clearly separated along the predictive x-axis (Figure 4A). Selecting the variables with a VIP-value above 1.5, and thereby most important for separating the two groups, resulted in 24 variables that were visualised in the loading plot (Figure 4B).

Additional correlations found between parameters not presented in text are shown in Supplementary Table S3.

## Discussion

Periodontal disease is associated with atherosclerotic CVD. Altered levels of several inflammation-related proteins in plasma indicated a systemic inflammatory response in the patients with periodontitis. By isolating LDL/VLDL and HDL particles and by using a proteomic approach, we hereby show that the plasma lipoproteins are altered towards an atherogenic form in patients with periodontitis.

We found altered levels of circulatory lipoproteins with decreased HDL (apoA-I), and increased LDL (apoB) and apoB/apoA-I ratio, which indicates a higher risk of CVD in the patient group [24,31]. In addition, high apoB/apoA-I ratio has been associated with early atherosclerosis [32]. The decreased circulatory levels of apoA-I found by the clinical chemistry test were reflected in the results from the nLC-MS/MS, but also the 2-DE showed lower amounts of pro-apoA-I as well as a glycated isoform of apoA-I. Alterations in HDL proteins and lipid constituents diminish the atheroprotective properties of HDL and thereby influence atherogenesis [21,33]. ApoA-I mediates many of the anti-atherogenic functions of HDL. It is required for cholesterol removal from cells (including macrophages) and has antioxidative and anti-inflammatory effects [21]. ApoA-I in HDL showed increased methionine oxidation which correlated to clinical periodontal parameters in the patient group. Methionine oxidations of apoA-I have been linked to impaired capacity to activate LCAT and ATP-binding cassette transporter 1-dependent cholesterol efflux, as well as reducing the ability to remove oxidised lipids [34,35]. Although the levels of LCAT in HDL of the patients was not significantly decreased compared with the controls, it is interesting to report that the levels of LCAT correlated negatively to pocket depth and to BOP%. Furthermore, higher LCAT and cholesteryl ester transfer protein activities after periodontal treatment support the hypothesis of reduced activation of these proteins and reversed cholesterol transport during periodontitis [36]. In addition, Grudyanov et al. (2017) [37] showed an association of changes in blood lipids, including a reduction in apoA-I with progression and development of chronic periodontal disease. In this study, HDL apoA-I expression in the 2-DE correlated negatively to pocket depth, especially to the deeper depths (>6 mm). Periodontitis-induced changes in HDL composition may impair its efflux capacity [36]. On the contrary, apoA-I in LDL of the patients showed decreased methionine oxidation, which may be explained by a consumption of antioxidative factors of HDL to protect LDL. Unpublished results by our group showed that patients with periodontitis expressed lower plasma antioxidant levels, and decreased lipid peroxidation (lipids of the plasma membrane of blood cells) 3 min after periodontal debridement [38]. After 20 min, the antioxidant levels dropped while the lipid peroxidation increased. The opposite was seen in the control group where the antioxidant levels dropped after 3 min, simultaneously with an increase in lipid peroxidation, suggesting a scavenging effect of the antioxidants on excess lipid peroxidation products. The results indicate that patients with periodontitis might be primed as the chronic inflammatory state in the oral cavity constantly triggers the immune system. This agrees with elevated total antioxidant levels detected in the patient group, protecting some of the lipids and proteins from oxidation. Another likely explanation is that modified LDL is cleared from the circulation by monocytes/macrophages, thereby reducing LDL apoA-I methionine oxidation in the patient group. Other studies have shown that oxidised lipids, such as oxidised phosphatidylcholines in lipoproteins, can cause HDL apoA-I oxidation at specific methionine residues [39,40]. This could be a mechanism for the apoA-I methionine oxidations and a protective function of apoA-I as a reducing agent.

PLTP promotes cholesterol efflux and reverses cholesterol transfer [41], and is essential for maintaining normal HDL levels. PLTP correlated positively to apoA-I and negatively to apoB/apoA-I ratio. ApoA-I increases the PLTP activity [41], which suggests that the decreased levels of apoA-I in HDL of the patients, as well as methionine oxidations, interfere in this regulation. Furthermore, increased PLTP activity is associated with less tissue damage caused by bacterial infection [24,33,41], further highlighting the importance of a normal regulation of the PLTP during infection. PLTP is reported to be a key mediator in the removal of lipopolysaccharides (a potent virulence factor of Gram-negative bacteria) to HDL followed by elimination in the liver. PLTP also exerts direct antibacterial effects, preventing the growth of Gram-negative bacteria [42]. This may be of importance, since the microflora in periodontitis is dominated by Gram-negative species, a finding also seen in the present study. Periodontal treatment has been shown to decrease the activity, but increase the level, of PLTP [36]. Thus, the decreased levels of PLTP in the patient group suggest that PLTP is consumed during periodontitis, and a reduced reverse cholesterol transport has been suggested to occur in the disease [20]. However, conflicting results of the association of the level and/or activity of PLTP to CVD, may depend on different infectious agents and the state of the inflammation, as well as differences in gender [43]. In our study, PLTP correlated positively to other inflammatory markers that were differently expressed in the study group, such as PON3, apoJ, apoM, and apoF, and negatively to apoA-IV, SAA4 and α-1-antichymotrypsin. ApoF is a lipid transfer inhibitor protein which has previously been shown to be positively associated with PLTP [30], corresponding to the results of the present study. ApoF also correlated positively to number of teeth and to PON1 and PON3.

PONs are vital components in HDL function due to their ability to hydrolyse lipid peroxides in LDL [21]. PON1 is the most studied of the three PON isoforms. In the present study, no difference in serum PON1 aryl esterase activity was found, which confirms the results of a previous study investigating PON1 activity in patients with periodontitis [44]. However, we found decreased HDL PON3 levels in the patient group. Draganov et al. [45] have shown that rabbit PON3 is able to effectively protect LDL from Cu^2+^-dependent oxidation, even more efficiently than PON1. In addition, PON3 protects LDL from mild oxidation and decreases levels of mildly oxidised LDL in cultures of human aortic endothelial cells [46]. Data suggest that PON3 possesses protective properties against atherogenesis. Studies in mice have shown that elevated levels of PON3 enhanced cholesterol efflux, which agrees with the positive correlation to PLTP found in the present study, as well as to the decreased LDL methionine oxidation, and increased antioxidant properties of HDL and of plasma, factors that promote slowing down of the atherosclerotic process [47].

Endothelial dysfunction might reflect alterations in the HDL proteome, including enrichment in inflammatory proteins, such as SAA, complement C3, apoC-III and/or a depletion of apoJ and apoA-IV [21]. In the present study, the patients showed decreased HDL apoA-IV, although this was not significant. ApoJ in HDL was decreased in the patient group, shown by both the 2-DE and nLC-MS/MS technique. ApoJ, also called clusterin, promotes cholesterol efflux from foam cells [48] and inhibits apoptosis of endothelial cells [49], which are potential anti-atherogenic functions. Interestingly, apoJ correlated with LCAT and PLTP expression in HDL in the present study, strengthening its involvement in cholesterol efflux.

ApoC-III was enriched in HDL from the patients. Increased apoC-III in HDL has shown a direct association with coronary heart disease and apoC-III is suggested to interfere with the atheroprotective function of HDL [21,24]. Riwanto et al. [49] observed reduced apoJ content of HDL, in combination with increased apoC-III, in patients with coronary artery disease. This leads to an impaired effect of HDL on endothelial antiapoptotic pathways and instead activation of pro-inflammatory signalling. ApoC-III is also an inhibitor of lipoprotein lipase, playing a vital role in the triglyceride metabolism [24]. ApoC-III correlated positively to pocket depth, BOP, GCF volume and SAA1, thus indicating an association with the inflammatory status of the patient group. An increased level of SAA1 is a risk factor for CVD [50]. SAA1 increased in HDL in the patient group shown by ELISA, 2-DE and nLC-MS/MS technique, though, the difference was not statistically significant between the study groups. SAA1 can displace apoA-I from HDL when concentrations of the acute phase protein are elevated, such as in coronary heart disease patients [51,52]. In this study, there was a negative correlation between total apoA-I and SAA1b in HDL (right SAA1 isoform on the 2-DE gel), and a negative correlation between apoA-I isoform (e) and SAA1 isoform (b) as well as total protein forms in LDL in nLC-MS/MS data. Replacement of apoA-I could be one possible mechanism of the reduced apoA-I levels, although this needs to be further evaluated. In addition, we found enrichment of the common SAA4 form, and decreased expression of one glycosylated SAA4 form, which suggests redistribution of the protein in HDL in periodontitis. This agrees with previous findings of reduced glycosylated forms of SAA4 in *P. gingivalis*-stimulated blood *ex vivo* [15]. The role of SAA4 is not well understood in human disease, however, all subtypes are associated *in vivo* with cholesterol control in tissues and plasma [53].

The patient group showed enriched apoA-II, a major structural protein in HDL, also binding competitively with apoA-I to PLTP [54]. The role of apoA-II is not clear, mouse models and epidemiologic studies report that apoA-II has pro- or anti-atherogenic properties, modulating lipid metabolism via multiple pathways depending on the content, structure and function of apoA-I in HDL [55]. An interaction between apoA-II and apoA-I is strengthened by the positive correlation with apoA-II shown in the present study. ApoA-II in HDL correlated to pocket depth, BOP, GCF volume, as well as to apoC-III and SAA1, and negatively to α-1 antitrypsin, indicating an association with periodontal and inflammatory status of the study individuals. ApoA-II in HDL was found to be important in discriminating between patients with periodontitis and controls by the multivariate analysis. Notably, apoA-II is also detected at increased levels in GCF of the patients with periodontitis compared with the controls (*P*<0.03; unpublished observations).

Increased levels of HDL-associated complement C3, a key player in innate immunity, has been identified in patients with CVD [49]. This is inconsistent with our finding of a down-regulation of C3 in HDL; however, it is important to bear in mind the complexity of the complement system. We have previously reported a down-regulation of HDL complement C3 in individuals with CVD, also exposed to persistent organic pollutants [30], which support the results of the present study. In addition, several periopathogens, including *P. gingivalis* and *P. intermedia* that were found in 40 and 50%, respectively, of the study patients, are very efficient at inactivating multiple complement components, including C3, C4 and C5, by proteolytic degradation. This is a way for bacteria to evade the host immune system and to protect the subgingival biofilm. *P. gingivalis* and *P. intermedia* are also resistant to complement-mediated lysis, which may lead instead to elimination of other bacteria competing for space and nutrients [56]. No correlation was found to identify periodontitis-associated bacteria, however, a negative correlation to *P. propionicus*, which is a part of the normal oral flora, was found which may suggest that this bacterium is sensitive to complement C-III-mediated killing. In addition, the subgingival amount of *P. propionicus* correlated negatively to *T. forsythia* that was the most frequently found periopathogen in the patients.

The OxyBlot of the LDL/VLDL gels revealed oxidation of α-2-HS-glycoprotein and α-1 antitrypsin in the patient group. α-2-HS-glycoprotein is a potent inhibitor of pathological vascular calcification [57], and α-1 antitrypsin is a serine protease inhibitor. Since neutrophil elastase, which has been found in atherosclerotic plaques [58], is able to modify apolipoproteins resulting in HDL destruction [59], the possibility is raised that the presence of α-1 antitrypsin in lipoprotein particles is of importance in the regulation of the inflammatory process during atherosclerosis. α-1 antitrypsin could be inactivated by oxidation, and damage to anti-proteinases induced by ROS is a hallmark of diseases, such as atherosclerosis and rheumatoid arthritis [60]. We have previously found that the *P. gingivalis* induce ROS in whole blood and in isolated neutrophil suspensions [14,15]. The level of α-1 antitrypsin found by nLC-MS/MS and one isoform in 2-DE was decreased in lipoproteins of the patient group, but not significantly compared with the controls. Oxidation of these anti-inflammatory proteins suggests reduced protective properties. The presence of oxidised albumin even in the control group is in-line with other studies [61]. α-1-antichymotrypsin is a protease inhibitor closely related to α-1 antitrypsin, antagonising overexpressed protease activity and also regulating ROS production [60]. In the present study, increased levels of α-1-antichymotrypsin in HDL correlated negatively to apoJ, PON1 and 3, as well as correlating positively to α-2-HS-glycoprotein, indicating an involvement of α-1-antichymotrypsin in antioxidative and vasculoprotective mechanisms, possible defending lipoprotein particles in the circulation and in the protease-rich plaque environment.

The LDL/VLDL proteome revealed increased expression of PAF-AH, also called lipoprotein-associated phospholipase A2, as well as apoL-1. Increased PAF-AH is a potential biomarker, and a strong independent risk factor, of CVD and is suggested to play a direct role in the atherosclerotic process. PAF-AH correlated negatively with apoB/apoA-I ratio in plasma. PAF-AH hydrolyses oxidised phospholipids in LDL, generating lysophosphatydyl choline, a molecule that exerts several proatherogenic effects and is important in human atherosclerotic plaque inflammation [62]. PAF-AH is also suggested to be an inflammatory marker for periodontitis [63] and Losche et al. [64] reported a decline in PAF-AH after periodontal therapy. This agrees with correlations of PAF-AH to pocket depth and volume of GCF of the study subjects in this study. In addition, PAF-AH was pointed out as a key component to discriminate patients from controls by the multivariate analysis.

ApoL-1 is foremost described as an HDL-associated apolipoprotein reported to exert several extra- and intracellular functions in host defence and haemostatic mechanisms, but is also involved in the transport and metabolism of lipids [65]. Recent research has shown that polymorphisms in apoL-1 are associated with an increased risk for CVD [66]. Although yet to be identified, this indicates a function for apoL-1 in CVD development. Duchateau et al. [67], suggested that apoL-1 association with HDL is dependent on pathways generating large HDL particles containing apoL and that formation of these particles involves activity of LCAT. Reduced LCAT activity and PLTP may promote the formation of larger HDL particles [41]. Therefore, we speculate that due to changed concentrations and activities of major HDL remodelling factors, probably generating less apoL-1-associated HDL particles during periodontitis, apoL-1 is redistributed to LDL/VLDL particles. However, this needs to be further clarified. ApoL-1 is reported as a component of VLDL and LDL [68], although the role of apoL-1 in these lipoproteins has not been explored. Furthermore, apoL is strongly induced in endothelial cells by tumour necrosis factor-α and interferon-γ, proinflammatory cytokines that are involved in CVD [10], and it is proposed that apoL-1 triggers apoptosis of endothelial cells [65]. Since apoptosis of endothelial cells is a crucial event in atherosclerosis and CVD [49], this suggests that apoL-1 could be involved in atherogenesis. In addition, apoL-1 correlated with BOP, apoB/apoA-I ratio and apoC-II further suggesting an involvement in the inflammation in periodontitis.

The 2D gels visualised excessive amounts of apoB fragments in LDL/VLDL from the patients. Both oxidative events and enzymatic degradation could cause ApoB-fragmentation [69]. Previously we found fragmentation of apoB-100 in LDL/VLDL induced by *P. gingivalis in vitro* [15,16]. *P. gingivalis*-induced fragmentation of apoB, and LDL from periodontitis patients, lead to increased uptake by macrophages, resulting in foam cell formation [20,70]. ApoB-100 interaction with the LDL-receptor is important for removing LDL and VLDL from the circulation. Interestingly, the LDL receptor in the liver does not recognise apoB-modified LDL, thereby accumulating LDL/VLDL in the circulation [71] and increased uptake by scavenger receptors and deposition in the vessel wall, eventually resulting in plaque formation. ApoB plays a critical role in lipoprotein transport, thus suggesting that the apoB fragmentation observed in the patient group may have an impact on LDL/VLDL deposition. In addition, we did not detect any apoE-fragmentation which we previously have shown to be induced by *P. gingivalis in vitro* [15]. If formed at all, these particles are likely cleared from the circulation by monocytes/macrophages.

In contrast with other studies [5], we did not find any differences in CRP levels between the study groups, probably because a highly sensitive CRP assay with a lower detection limit was not used and only females were included. Fasting glucose levels were increased in patients with periodontitis, which is in-line with previous studies [5]. ApoA-I correlated to plasma thyrotropin, which is supported by other studies [72], and thyroid hormones can influence lipid metabolism [24].

The multivariate model investigates which factors are responsible for separating the study groups. It was also used as a complementary test to reduce false discovery rate that could be a problem with the considerable amount of data. The model showed a clear separation between the two groups (score plot) where the controls showed a tight grouping, while the patient group showed a larger variation, as can be seen by spreading along the y-axis. Due to a high number of variables, only those with a VIP-value over 1.5 were visualised in the loading plot. Clinical periodontal parameters, such as periodontal pocket depth, BOP and the volume of GCF, as well as amount of some periopathogens extracted from periodontal pockets discriminate the study groups. These are standard parameters used for periodontitis diagnosis. ApoB/apoA-I ratio correlated to the patient group. Interestingly, PAF-AH in LDL and apoA-I, apoJ and apoA-II in HDL were found important for discriminating between the groups, as well as SAA4.

Confounders in our study may be smoking and obesity. BMI correlated negatively with apoA-I levels as well as to apoJ in HDL and was also found to discriminate the study groups in the multivariate modelling. However, it is demonstrated that chronic periodontitis and obesity, jointly or individually, are associated with undesirable proatherogenic lipid profiles [73]. No correlations were found to smoking, except a negative correlation of one apoA-I isoform found in 2D gels and Ig κ chain C region in HDL registered in nLC-MS/MS analysis. A limitation of the present study is the small study group and inclusion of only females, it is likely that more differences in the HDL and LDL/VLDL proteome would have been found in a larger study population and with inclusion of males. Atherosclerosis, and oxidative stress, are associated with reduced levels of endogenous NO, which is important in regulating the vascular tone. The serum levels of the NO metabolite nitrate were reduced in the patient group, although not significantly, which is in-line with other studies showing reduced levels of NO metabolites in periodontitis but only in male population [74]. Gender is an important factor to consider in future studies of the relation between periodontitis and CVD.

An important consideration not analysed in the present study is the lipid content of the lipoproteins, which have a strong influence on their properties in the context of atherosclerosis. Other studies have shown that LDL tend to be smaller and denser, and the levels of LDL-cholesterol, triglycerides and triacylglycerols tend to be elevated in periodontitis [20]. Density-gradient ultracentrifugation is the most common method for isolation of lipoprotein particles, which has the drawback with a risk of some overlap between the density/size ranges of lipoprotein classes. At the same time, this overlap may also reflect the *in vivo* situation. The short-spin ultracentrifugation was used to minimise the loss and exchange of proteins, and two repeated centrifugation steps, to eliminate as many contaminants as possible.

The present study is primarily observational and functional studies of lipoprotein modifications and involved mechanisms is essential for understanding the link between periodontal and CVD.

In conclusion, patients with periodontitis have an altered plasma lipoprotein profile, including altered levels and structural modifications of both HDL and LDL/VLDL towards a proinflammatory atherogenic form, which supports a role of modified plasma lipoproteins as central factors in the link between periodontal and CVD.

## Perspectives

- Periodontitis is associated with atherosclerotic CVD and modified lipoproteins are central in atherogenesis, therefore the present study analyses the lipoprotein particles in periodontitis.
- Periodontitis patients exhibited an atherogenic lipoprotein profile; reduced anti-inflammatory proteins on HDL and an increased proinflammatory LDL expression.
- The study illuminates modified lipoproteins as a potential link between periodontitis and atherosclerosis, and will aid future studies on mechanisms and a causal relationship.

## Supporting information

**Supplemental Table 1.** Protein quantities as found by 2-DE. Values are ppm of total 2-D gel staining.

**Supplemental Table 2.** Protein quantities as obtained through nLC-MS/MS. Values are MaxQuant LFQ intensities as obtained through utilized software.

**Supplemental Table 3.** Correlations found between parameters analysed, not presented in Result section.

**Supplemental Table 4.** The concentrations of 71 cytokines, chemokines and growth factors in plasma of patients with periodontitis and healthy controls, analysed by a multiplex immunoassay. * indicates significant (P<0.05) difference between controls and patients. Mann-Whitney U test was used for data that was not normally distributed and Students T-test for normally distributed samples.

## Abbreviations

Apo: apolipoprotein
BOP: bleeding on probing
CRP: C-reactive protein
CVD: cardiovascular disease
GCF: gingival crevicular fluid
HDL: high-density lipoprotein
IAA: iodoacetamide
IPG: immobilized pH gradient
LCAT: phosphatidylcholine-sterol acyltransferase
LDL: low-density lipoprotein
MCH: mean corpuscular haemoglobin
MCHC: MCH concentration
nLC-MS/MS: nanoliquid tandem MS
OPLS-DA: orthogonal partial least square discriminate analysis
PAF-AH: platelet-activating factor acetylhydrolase
PCA: principal component analysis
PLTP: phospholipid transfer protein
PON1: serum paraoxonase/arylesterase 1
PON3: serum paraoxonase/lactonase 3
PPD: probing pocket depth
ROS: reactive oxygen species
SAA: serum amyloid A
VIP: variable importance in projection
VLDL: very LDL
2-DE: 2D gel electrophoresis
