# Supplementary material for: Modified lipoproteins in periodontitis: a link to cardiovascular disease?
Source: Biosci Rep. 2019 Mar 26;39(3):BSR20181665. doi: 10.1042/BSR20181665 (PMC6434390; doi:10.1042/BSR20181665)
Supplement: Supplementary file 1 [file bsr-39-bsr20181665_Supp1.pdf]

Supplemental Table 1. Protein quantities as found by 2-DE. Values are ppm of total 2-D gel staining.

Protein spot	SSP-ID	Control	Patient	P-value MW
LDL/VLDL				
A1ATa	1505	208,2 (75,7 - 963,4)	177,75 (18 - 966,7)	0.464215
A1ATb	1501	1059,7 (339,6 - 1932,4)	539,25 (20,2 - 2022,9)	0.261742
A1ATc	1502	1048,4 (472,7 - 1921,2)	670,8 (22,4 - 3804,7)	0.261742
A1ATd	1503	295,1 (170,4 - 1052,9)	169,3 (0 - 543,3)	0.157053
A1ATe	1504	203,3 (86,2 - 303,1)	104,35 (26,6 - 873,8)	0.591444
A1AT-Total		3021 (2065,6 - 5363,3)	1694,95 (189,8 - 6772,5)	0.222513
ApoA1a	1109	9590,8 (807,6 - 29722,6)	4043,5 (851,1 - 15461,6)	0.406814
ApoA1b	1110	8059,5 (4462,5 - 16640,9)	10875,15 (4798 - 29122,4)	0.157053
ApoA1c	2003	7336,3 (4258,9 - 18360,5)	7976,2 (1615,6 - 35625)	0.406814
ApoA1d	2110	3886,2 (2458,9 - 6311,4)	1494,1 (60,1 - 6087,3)	0.012827
ApoA1e	2109	8249,3 (6425,7 - 12065,9)	4532,7 (1102,8 - 11385,6)	0.009706
ApoA1-Total		35076,2 (19347,8 - 81257)	37148,65 (25005,1 - 67717,9)	0.883618
ApoA2	15	4765,2 (374,5 - 6835,8)	960,25 (30 - 6723,2)	0.071011
ApoA4a	1309	389,2 (165,6 - 1980,2)	605 (22,4 - 1109,8)	0.80725
ApoA4b	1308	1138,4 (174,8 - 1912,4)	1046,55 (31,7 - 2243,4)	0.883618
ApoA4-Total		1630,6 (763,2 - 2421,3)	1570,95 (75,5 - 2930,2)	0.883618
ApoBfragment	9304	3126,3 (1380,3 - 15813,1)	2117,8 (214,2 - 17408,9)	0.464215
ApoC2	18	3535,7 (207,3 - 12552)	3448,8 (513,3 - 19435,7)	0.961083
ApoC3a	16	1977,5 (1094,7 - 11945,1)	4972,45 (310,6 - 23581,8)	0.406814
ApoC3b	17	2217,1 (447,7 - 16235,7)	3013,3 (99,3 - 25374,5)	0.961083
ApoC3c	11	4265,1 (54,1 - 15618)	3520,35 (80,2 - 23485,5)	0.961083
ApoC3d	12	5551,9 (243,2 - 10555,5)	3503,85 (1014,2 - 14836,1)	0.525863
ApoC3e	13	1890,3 (78 - 9709,6)	2113,8 (76,2 - 6617,3)	0.883618
ApoC3f	14	132,2 (12,4 - 3411,2)	63,35 (19,6 - 1961,6)	0.883618
ApoC3-Total		30418,7 (11542,5 - 32461,5)	33030,85 (10004,3 - 66555,5)	0.464215
apoEa	2303	557 (83,6 - 1785,4)	500,75 (24,1 - 7997,9)	0.66055
apoEb	2214	4604,1 (610 - 9895,3)	3042,65 (1901,6 - 15379,4)	0.883618
apoEc	2302	7146,2 (1920 - 20945,6)	3981,8 (1280 - 13923,5)	0.406814
apoEd	2212	5049,2 (2393,9 - 18381,1)	2827,5 (1687,4 - 15123,9)	0.261742
apoEe	3205	2549,7 (142,3 - 18803,4)	139,25 (0 - 8272,3)	0.03589
ApoE-Total		23124,5 (7398,9 - 46423,6)	18145,85 (6957,9 - 31938,7)	0.157053
apoJa	1310	256,8 (46,6 - 1388,5)	101,2 (5,3 - 1685,8)	0.353873
apoJb	1307	2662,9 (74,1 - 5018,6)	2858,2 (37,3 - 4138,3)	0.80725
apoJc	1306	1957,3 (465,5 - 3212,9)	1973,55 (29,1 - 5926,9)	0.961083
apoJd	1305	671,7 (176,1 - 1706,9)	751 (24,7 - 2858,7)	0.883618
ApoJ-Total		5320,6 (889 - 10369,7)	5684,8 (96,4 - 14609,7)	0.961083
apoMa	1206	4098,8 (1110,7 - 5639,2)	4144,55 (616,2 - 6563,3)	0.406814
apoMb	1207	2660,7 (271,7 - 7468,7)	2711,8 (94,8 - 13989,2)	0.961083
apoMc	2210	11399,9 (2651,4 - 15329,9)	8527,8 (3424,2 - 22213,5)	0.961083
apoMd	2211	10065,3 (2476,9 - 13292,1)	9526,3 (5632,5 - 16487,5)	0.525863

apoMe	3204	2332,2 (22 - 3885,3)	2104,35 (96,2 - 4715,6)	0.80725
apoMf	3104	4155,9 (603,3 - 5170,7)	3266,25 (416 - 7794,8)	0.961083
ApoM-Total		37183,8 (11056,9 - 41236,8)	33039,25 (13710,3 - 56756,5)	0.591444
SAA1a	3002	103,3 (2,5 - 1215,2)	5,1 (0 - 108,8)	0.045437
SAA1b	4001	51,3 (11,3 - 397,5)	45,55 (0 - 445,3)	0.558185
SAA1-Total		174,5 (19,6 - 1294,8)	84,9 (0 - 455,5)	0.08767
SAA4a	6002	265,3 (65,9 - 574,6)	56,8 (18,1 - 3594,8)	0.222513
SAA4b	7003	1295,4 (87,4 - 5202,4)	2466,5 (114,3 - 14179,1)	0.353873
SAA4c	8003	3016,5 (164,9 - 6408,1)	4163,45 (133,9 - 17806)	0.591444
SAA4d	6003	23,5 (0 - 156,3)	36,55 (0 - 640,8)	0.922258
SAA4e	7004	133,5 (0 - 1422,5)	231,55 (50,4 - 5862,6)	0.187684
SAA4f	8004	1835,1 (74,1 - 7489)	2412,8 (256,2 - 16001,5)	0.464215
SAA4-Total		10277,6 (446,6 - 14788,5)	11243,05 (617,1 - 45427,1)	0.353873
TTR	3001	280,3 (62,7 - 1084,6)	221,45 (15,5 - 1018,1)	0.353873
HDL				
Albumin a	5904	489,7 (247,2 - 1041)	319,15 (53,9 - 1006,4)	0.347746
Albumin b	5902	651,2 (244,9 - 1564,1)	788,8 (111,5 - 1259,5)	0.967436
Albumin c	5903	796 (260,8 - 1466,1)	691,65 (223,9 - 1766,2)	0.838257
Albumin d	5901	1735,4 (871,2 - 5821,8)	2193,15 (369,4 - 3915,4)	0.653379
Albumin e	6903	2417,1 (1656,5 - 5029)	3708,2 (570,2 - 6290,8)	0.713303
Albumin f	6902	5680,4 (3725 - 14316,7)	5803,95 (1113,8 - 12241,7)	0.713303
Albumin g	6901	9752,9 (3366,4 - 17563,3)	5327,35 (1595,4 - 16356,5)	0.270345
Albumin Total		22703,1 (13662,7 - 41749,5)	24495,15 (4038,1 - 34204,3)	0.775052
alpha1-ATa	1805	477,6 (205,3 - 933,6)	538,65 (81,4 - 1090,6)	0.967436
alpha1-ATb	1806	1165 (487,6 - 1936,9)	1518,7 (232,7 - 2601)	0.713303
alpha1-ATc	1807	1610,6 (676,2 - 2084,1)	1685,6 (273,7 - 3924,9)	0.902523
alpha1-ATd	2804	671,6 (120,2 - 1458,5)	828,2 (520,8 - 1236,5)	0.902523
alpha1-ATe	2803	1801,5 (573 - 4017,3)	1295,45 (338,6 - 2296,4)	0.177911
alpha1-ATf	3801	1483,4 (865,5 - 4078,3)	1590,1 (400,9 - 2751,4)	0.902523
alpha1-AT Total		7755,9 (4469,9 - 10046,3)	7737,9 (2869,4 - 12238)	0.713303
Alpha-2-HS-glycoprot a	803	88,4 (2 - 275,4)	56,65 (3,4 - 380,8)	0.838257
Alpha-2-HS-glycoprot b	901	241,4 (10 - 400,4)	194,9 (11,2 - 492,3)	0.838257
Alpha-2-HS-glycoprot c	902	146,6 (93,3 - 595,4)	362,35 (5,1 - 632,9)	0.236446
Alpha-2-HS-glycoprot d	903	164,5 (67,8 - 385,3)	184,25 (63,5 - 611,9)	0.347746
Alpha-2-HS-glycoprot e	804	49 (1,6 - 267,5)	112,7 (3,1 - 273,2)	0.487669
Alpha-2-HS-glycoprot Total		585,3 (283,8 - 1696,7)	1107,7 (128,4 - 2155,1)	0.653379
ApoA-I	2901	545014,8 (502238 - 621869,3)	518639,1 (432613,1 - 649632,6)	0.307435
ApoA-Iglyca	2912	845,8 (85,5 - 3411,2)	1907,9 (159,8 - 2897,9)	0.391268
ApoA-IGlycb	2905	2840,3 (1025,2 - 4417,6)	1503,55 (243,6 - 2486,4)	0.030487
ApoA-IGlycc	2906	2638,5 (1622,6 - 4593,6)	3134,55 (1508,9 - 4087,5)	0.902523
ApoA-IGlycd	2911	135,5 (38,6 - 471,3)	389 (17,4 - 844,3)	0.236446

ApoA-IGlyce	2910	1201,7 (507,8 - 2304,9)	1566,9 (68,5 - 2855,4)	0.540292
ApoA-IGlycf	2909	1774,9 (807,5 - 2447,3)	2372,3 (780,3 - 2944,7)	0.094167
ApoA-IGlycTotal		10413,4 (6905,5 - 12516,7)	10783,6 (3451,6 - 14652)	0.653379
ApoA-II	1	4009,5 (464,9 - 7558,3)	3668,35 (47,1 - 7225,7)	0.775052
ApoA-IVa	2917	514,3 (172,5 - 669,6)	441,45 (204,2 - 600,9)	0.347746
ApoA-IVb	2918	1682,5 (688,2 - 2647,2)	1602 (949,7 - 2949,3)	0.967436
ApoA-IVc	2919	724,6 (383,6 - 1609,1)	586,45 (136,6 - 1089,8)	0.205668
ApoA-IVTotal		3024,6 (1543,4 - 4342,4)	2624,3 (1677,3 - 3686,8)	0.347746
ApoC-I	9922	682,8 (106,8 - 1289,5)	1216,55 (23,4 - 2171,9)	0.153043
ApoC-II	103	10109 (6862,1 - 16177,6)	8520,15 (3311,6 - 16464,4)	0.177911
ApoC-IIIa	101	9345,2 (2679,1 - 12921,4)	8925,05 (1848,4 - 15062,9)	0.540292
ApoC-IIIb	102	10518,2 (2407,3 - 26711,6)	10208,6 (6254,6 - 17318,4)	0.902523
ApoC-IIITotal		20566,7 (5086,4 - 39527,3)	19485,2 (13082,5 - 32116,6)	0.967436
ApoEa	2907	3976 (1121,3 - 13480,3)	3687,1 (1207,8 - 9592,2)	0.838257
ApoEb	2908	8153,6 (927,6 - 17493,5)	6888,35 (3001,1 - 16332,9)	0.713303
ApoEc	3904	8757 (1586,4 - 12175,5)	8698,25 (2737,4 - 12193,3)	0.713303
ApoEd	3905	2720,4 (493,4 - 6956,5)	618,3 (12,9 - 8345)	0.130912
ApoE-Total		26909,6 (8965,3 - 44881,3)	20375,75 (12353 - 37401,9)	0.775052
ApoJa	2913	727,5 (55,6 - 1089,1)	391,3 (106,6 - 595,3)	0.019965
ApoJb	2914	580,8 (437,6 - 793)	364,45 (12,9 - 539,2)	0.00052
ApoJc	2915	431,9 (23,9 - 993,7)	360,1 (131,1 - 503,9)	0.270345
ApoJTotal		1683,3 (517,1 - 2851,5)	1027,7 (307,5 - 1449,8)	0.003749
ApoL-1a	5701	144,8 (69,3 - 620,4)	201,65 (123,7 - 1210,8)	0.205668
ApoL-1b	6701	285,2 (3,6 - 546,5)	343,4 (1,2 - 747,1)	0.307435
ApoL-1c	6702	437,8 (195 - 886,6)	540,3 (6,7 - 1623,5)	0.487669
ApoL-1Total		819 (605,5 - 1580,3)	1080,5 (508,2 - 2823,6)	0.391268
ApoMa	2904	2185 (772,4 - 3362,7)	1713,5 (1263,4 - 3560,8)	0.347746
ApoMb	2902	4769,9 (3838,5 - 5609,5)	4633,85 (3223,5 - 5085,9)	0.391268
ApoMc	3901	2105,6 (1519,2 - 3309,3)	1846,4 (1118,3 - 2840,5)	0.437943
ApoMTotal		8929,5 (6254,9 - 10945,5)	8757,4 (6186,5 - 10338,4)	0.391268
PON1a	802	424,2 (57,4 - 1118,1)	359,05 (77,1 - 724,2)	0.347746
PON1b	1803	57,6 (16,3 - 1311,7)	166,85 (14 - 349,9)	0.270345
PON1c	1804	151,3 (17,7 - 451,7)	108,7 (38,6 - 407,4)	0.902523
PON1Total		688,7 (238 - 2293,2)	776,2 (223,9 - 1082,1)	0.713303
SAA1a	9911	1883,3 (768,1 - 6916,4)	3423,9 (1658,1 - 4571,9)	0.153043
SAA1b	9912	1455,6 (331,8 - 4606,2)	2822,75 (445,4 - 6141,4)	0.307435
SAA2a	9918	7,7 (0,8 - 836,8)	32,95 (2,5 - 1639,5)	0.391268
SAA2b	9920	285,7 (1,4 - 547,4)	243,65 (2,8 - 1053,1)	0.902523
SAA4a	9903	4545,4 (2798,6 - 6297,6)	2625,05 (1542 - 4267,7)	0.007964
SAA4b	9904	7995,8 (2886,5 - 9950,9)	6790,15 (4,9 - 11565,4)	0.487669
SAA4c	9905	9182,8 (4905,4 - 18721,5)	8519,1 (2847,4 - 14565,8)	0.713303
SAA4d	9906	1030,1 (110,2 - 1864,4)	934,1 (72 - 2292,7)	0.775052
SAA4e	9907	4198,9 (805 - 5346,1)	3790,35 (11 - 9457,8)	0.595611
SAA4f	9902	20955,4 (9645,1 - 31165,9)	32117,75 (21287 - 46488,1)	0.006233
SAA4Total		49481,5 (38691,5 - 83433,6)	63415,55 (47328,5 - 77717,1)	0.066193

TTR	9913	1690 (897,7 - 2966,2)	1346,2 (472,8 - 3282,4)	0.540292

MW = Mann Whitney U-test

Supplemental Table 2. Protein quantities as obtained through nLC-MS/MS. Values are MaxQuant LFQ intensities as obtained through utilized software.

Protein	Gene name	Control	Patient	P-value MW
LDL/VLDL				
Alpha-1-acid glycoprotein 2	ORM2	1,2E+6 (8,8E+5 - 1,6E+6)	1,1E+6 (5,1E+5 - 1,6E+6)	0.312322
Angiomotin-like protein 1	AMOTL1	7,5E+5 (4,2E+5 - 3,2E+6)	1,1E+6 (5,5E+5 - 3,0E+6)	0.720985
Apolipoprotein A-I	APOA1	2,0E+9 (9,4E+8 - 2,8E+9)	9,1E+8 (1,4E+8 - 1,8E+9)	0.007964
Apolipoprotein A-II	APOA2	3,3E+8 (2,3E+8 - 4,9E+8)	4,1E+8 (2,7E+8 - 6,6E+8)	0.307435
Apolipoprotein A-IV	APOA4	4,5E+6 (1,7E+6 - 1,0E+7)	4,2E+6 (6,7E+5 - 8,7E+6)	0.391268
Apolipoprotein B-100	APOB	1,7E+10 (1,2E+10 - 2,1E+10)	1,6E+10 (1,2E+10 - 2,0E+10)	0.775052
Apolipoprotein C-I	APOC1	7,0E+7 (4,1E+7 - 1,0E+8)	7,3E+7 (3,6E+7 - 1,3E+8)	0.653379
Apolipoprotein C-II	APOC2	2,8E+8 (1,0E+8 - 3,6E+8)	3,8E+8 (6,5E+7 - 8,1E+8)	0.130912
Apolipoprotein C-III	APOC3	5,3E+8 (3,0E+8 - 1,0E+9)	8,1E+8 (2,2E+8 - 1,5E+9)	0.205668
Apolipoprotein C-IV	APOC4	2,0E+7 (1,1E+7 - 5,2E+7)	2,7E+7 (7,1E+6 - 5,3E+7)	0.653379
Apolipoprotein D	APOD	1,8E+8 (1,3E+8 - 3,8E+8)	2,5E+8 (1,6E+8 - 3,1E+8)	0.094167
Apolipoprotein E	APOE	6,4E+8 (2,5E+8 - 1,1E+9)	5,7E+8 (1,7E+8 - 7,7E+8)	0.391268
Apolipoprotein F	APOF	3,5E+7 (2,5E+7 - 5,5E+7)	3,7E+7 (2,2E+7 - 9,1E+7)	0.967436
Apolipoprotein L1	APOL1	1,2E+7 (8,0E+6 - 2,4E+7)	3,7E+7 (3,7E+6 - 1,5E+8)	0.024745
Apolipoprotein M	APOM	6,5E+7 (5,3E+7 - 1,1E+8)	8,9E+7 (5,7E+7 - 1,5E+8)	0.055015
Apolipoprotein(a)	LPA	1,4E+8 (7,8E+6 - 1,4E+9)	4,8E+7 (7,8E+5 - 9,5E+8)	0.236446
Calmodulin-like protein 5	CALML5	4,6E+5 (2,8E+5 - 7,3E+5)	4,0E+5 (2,3E+5 - 8,5E+5)	0.749119
Clusterin	CLU	9,5E+6 (6,9E+6 - 1,6E+7)	1,0E+7 (5,7E+6 - 1,8E+7)	0.595611
Complement C3	C3	6,8E+7 (4,6E+7 - 8,2E+7)	7,6E+7 (3,6E+7 - 1,1E+8)	0.391268
Complement C4-A	C4A	4,0E+7 (2,8E+7 - 7,7E+7)	4,8E+7 (2,1E+7 - 7,9E+7)	0.838257
Complement C4-B	C4B	1,9E+6 (8,8E+5 - 5,6E+6)	2,9E+6 (8,8E+5 - 3,5E+6)	0.385419
Complement factor H-related protein 4	CFHR4	1,5E+6 (8,6E+5 - 5,3E+6)	1,4E+6 (6,2E+5 - 3,7E+6)	0.784191
Dermcidin	DCD	3,9E+6 (2,4E+6 - 9,1E+6)	3,0E+6 (1,4E+6 - 9,4E+6)	0.540292
Fibrinogen alpha chain	FGA	1,4E+5 (9,4E+4 - 1,7E+5)	1,9E+5 (1,0E+5 - 2,5E+5)	0.100413
Haptoglobin-related protein	HPR	2,2E+6 (6,9E+5 - 8,9E+6)	2,0E+6 (9,7E+5 - 6,7E+6)	0.929637
Ig alpha-1 chain C region	IGHA1	9,5E+6 (4,4E+5 - 1,1E+7)	8,6E+6 (3,8E+5 - 1,7E+7)	0.859819
Ig gamma-1 chain C region	IGHG1	6,2E+5 (4,0E+5 - 8,2E+5)	8,3E+5 (4,1E+5 - 1,3E+6)	0.032278
Ig kappa chain C region	IGKC	3,8E+6 (1,3E+6 - 6,6E+6)	3,2E+6 (5,8E+5 - 6,0E+6)	0.5365
Ig lambda-3 chain C regions	IGLC3	4,3E+6 (2,1E+6 - 5,6E+6)	3,8E+6 (1,1E+6 - 5,6E+6)	0.307435
Platelet-activating factor acetylhydrolase	PLA2G7	3,3E+6 (2,4E+6 - 5,3E+6)	5,7E+6 (2,5E+6 - 7,5E+6)	0.012764
Prenylcysteine oxidase 1	PCYOX1	2,4E+7 (1,7E+7 - 2,7E+7)	2,8E+7 (1,8E+7 - 5,2E+7)	0.153043
Protein MENT	MENT	6,6E+5 (4,0E+5 - 8,4E+5)	5,3E+5 (2,0E+5 - 7,3E+5)	0.27159
Serum albumin	ALB	4,7E+7 (3,1E+7 - 7,1E+7)	4,7E+7 (2,9E+7 - 1,3E+8)	0.653379
Serum amyloid A-1 protein	SAA1	9,9E+5 (3,1E+5 - 5,6E+6)	2,0E+6 (9,5E+5 - 3,3E+6)	0.142632
Serum amyloid A-4 protein	SAA4	4,1E+7 (2,5E+7 - 7,4E+7)	5,9E+7 (2,5E+7 - 1,5E+8)	0.270345
Serum paraoxonase/arylesterase 1	PON1	2,7E+7 (1,5E+7 - 4,4E+7)	5,9E+7 (1,4E+7 - 1,8E+8)	0.066193
Tissue factor pathway inhibitor	TFPI	1,6E+6 (7,1E+5 - 3,3E+6)	1,0E+6 (7,1E+5 - 2,5E+6)	0.470487
Vesicular integral-membrane protein VIP36	LMAN2	1,7E+5 (9,3E+4 - 3,0E+5)	2,1E+5 (1,4E+5 - 4,3E+5)	0.689157

Vitronectin	VTN	7,1E+5 (4,2E+5 - 2,4E+6)	1,3E+6 (6,6E+5 - 2,5E+6)	0.100413
HDL				
Alpha-1-acid glycoprotein 1	ORM1	2,0E+6 (1,1E+6 - 6,4E+6)	3,0E+6 (9,9E+5 - 5,2E+6)	0.723932
Alpha-1-acid glycoprotein 2	ORM2	6,8E+5 (2,5E+5 - 1,9E+6)	7,9E+5 (3,9E+5 - 1,1E+6)	0.961627
Alpha-1-antichymotrypsin	SERPINA3	5,9E+5 (3,6E+5 - 1,3E+6)	1,1E+6 (4,8E+5 - 1,4E+6)	0.042844
Alpha-1-antitrypsin	SERPINA1	2,1E+7 (7,3E+6 - 3,3E+7)	9,1E+6 (3,7E+6 - 3,3E+7)	0.094167
Alpha-1B-glycoprotein	A1BG	4,9E+5 (3,2E+5 - 7,9E+5)	7,1E+5 (4,0E+5 - 8,3E+5)	0.235334
Alpha-2-HS-glycoprotein	AHSG	3,5E+6 (6,0E+5 - 6,2E+6)	4,4E+6 (5,8E+5 - 1,2E+7)	0.713303
Antithrombin-III	SERPINC1	5,8E+5 (3,7E+5 - 1,1E+6)	5,8E+5 (4,1E+5 - 8,9E+5)	0.943057
Apolipoprotein A-I	APOA1	8,5E+9 (7,7E+9 - 1,0E+10)	1,0E+10 (7,9E+9 - 1,5E+10)	0.07918
Apolipoprotein A-II	APOA2	1,9E+9 (1,6E+9 - 2,5E+9)	2,7E+9 (2,1E+9 - 3,4E+9)	0.0022
Apolipoprotein A-IV	APOA4	2,3E+7 (1,5E+7 - 3,2E+7)	1,8E+7 (1,1E+7 - 3,8E+7)	0.595611
Apolipoprotein B-100	APOB	7,0E+6 (8,6E+5 - 1,9E+7)	6,9E+6 (1,0E+6 - 1,2E+7)	0.824212
Apolipoprotein C-I	APOC1	1,0E+8 (7,1E+7 - 1,6E+8)	1,2E+8 (8,1E+7 - 2,4E+8)	0.177911
Apolipoprotein C-II	APOC2	1,1E+8 (5,7E+7 - 1,4E+8)	1,0E+8 (7,2E+7 - 2,6E+8)	0.902523
Apolipoprotein C-III	APOC3	5,0E+8 (4,6E+8 - 6,0E+8)	7,0E+8 (4,8E+8 - 1,4E+9)	0.012764
Apolipoprotein C-IV	APOC4	5,6E+6 (4,1E+6 - 9,5E+6)	6,7E+6 (4,7E+6 - 2,9E+7)	0.307435
Apolipoprotein D	APOD	2,9E+8 (1,9E+8 - 3,7E+8)	2,3E+8 (1,4E+8 - 5,1E+8)	0.307435
Apolipoprotein E	APOE	2,6E+8 (1,0E+8 - 6,5E+8)	2,8E+8 (1,6E+8 - 5,1E+8)	0.775052
Apolipoprotein F	APOF	2,8E+7 (1,7E+7 - 5,5E+7)	1,7E+7 (1,1E+7 - 4,2E+7)	0.037337
Apolipoprotein L1	APOL1	3,5E+7 (1,5E+7 - 5,3E+7)	2,9E+7 (2,5E+7 - 3,9E+7)	0.967436
Apolipoprotein M	APOM	1,1E+8 (8,1E+7 - 1,5E+8)	7,7E+7 (4,6E+7 - 1,7E+8)	0.153043
Apolipoprotein(a)	LPA	3,9E+6 (2,0E+5 - 1,3E+7)	8,3E+5 (2,2E+5 - 2,7E+6)	0.138363
Beta-2-glycoprotein 1	APOH	1,3E+6 (1,4E+5 - 3,5E+6)	8,8E+5 (2,6E+5 - 4,4E+6)	0.874826
Cathelicidin antimicrobial peptide	CAMP	1,1E+6 (3,5E+5 - 1,5E+6)	8,8E+5 (4,2E+5 - 1,8E+6)	1
Clusterin	CLU	5,2E+6 (3,9E+6 - 7,5E+6)	2,8E+6 (1,8E+6 - 6,9E+6)	0.012764
Complement C3	C3	2,7E+6 (5,3E+5 - 2,3E+7)	1,0E+6 (8,7E+5 - 2,9E+6)	0.037337
Complement C4-A	C4A	2,6E+6 (9,0E+5 - 3,9E+7)	2,7E+6 (3,2E+5 - 4,4E+6)	0.347746
Complement C4-B	C4B	7,6E+5 (3,9E+5 - 5,5E+6)	8,1E+5 (3,2E+5 - 1,5E+6)	0.830324
Dermcidin	DCD	6,1E+5 (3,9E+5 - 1,0E+6)	5,3E+5 (2,4E+5 - 1,1E+6)	0.494837
Glycophorin-A	GYPA	3,2E+5 (2,9E+5 - 5,5E+5)	3,0E+5 (2,6E+5 - 3,6E+5)	0.240956
Haptoglobin-related protein	HPR	7,5E+5 (3,3E+5 - 1,4E+6)	6,0E+5 (4,1E+5 - 1,2E+6)	0.768278
Hemopexin	HPX	3,8E+6 (6,1E+5 - 9,8E+6)	6,3E+6 (3,9E+5 - 8,0E+6)	0.531668
Ig alpha-1 chain C region	IGHA1	5,2E+5 (3,9E+5 - 1,1E+6)	6,5E+5 (3,6E+5 - 1,7E+6)	1
Ig gamma-1 chain C region	IGHG1	4,6E+6 (2,9E+6 - 8,0E+6)	3,1E+6 (2,8E+5 - 7,7E+6)	0.270149
Ig kappa chain C region	IGKC	2,1E+6 (1,6E+6 - 3,9E+6)	1,7E+6 (3,2E+5 - 3,1E+6)	0.037337
Ig lambda-1 chain C regions	IGLC1	5,1E+5 (2,8E+5 - 8,2E+5)	7,5E+5 (3,4E+5 - 9,3E+5)	0.143853
Kininogen-1	KNG1	3,1E+5 (1,9E+5 - 7,2E+5)	4,5E+5 (1,2E+5 - 1,3E+6)	0.701478
Phosphatidylcholine-sterol acyltransferase	LCAT	3,8E+6 (1,7E+6 - 6,8E+6)	2,0E+6 (1,5E+6 - 4,4E+6)	0.07505
Phospholipid transfer protein	PLTP	3,5E+6 (2,3E+6 - 4,5E+6)	1,8E+6 (7,8E+5 - 4,0E+6)	0.030487
Plasma protease C1 inhibitor	SERPING1	3,1E+5 (2,0E+5 - 6,7E+5)	3,6E+5 (2,7E+5 - 7,7E+5)	0.53087
Prenylcysteine oxidase 1	PCYOX1	2,5E+6 (8,2E+5 - 3,6E+6)	1,9E+6 (6,1E+5 - 4,0E+6)	0.437943
Protein AMBP	AMBP	3,9E+5 (2,5E+5 - 5,5E+5)	4,0E+5 (3,0E+5 - 4,7E+5)	0.701478

Serotransferrin	TF	2,1E+6 (3,6E+5 - 3,4E+6)	3,3E+6 (2,1E+5 - 4,6E+6)	0.205668
Serum albumin	ALB	1,0E+9 (5,3E+8 - 1,6E+9)	1,2E+9 (4,5E+8 - 2,4E+9)	0.967436
Serum amyloid A-1 protein	SAA1	7,1E+6 (4,1E+5 - 3,6E+7)	9,4E+6 (2,3E+6 - 2,8E+7)	0.487669
Serum amyloid A-4 protein	SAA4	1,3E+8 (8,5E+7 - 2,5E+8)	1,7E+8 (9,1E+7 - 2,3E+8)	0.094167
Serum paraoxonase/arylesterase 1	PON1	1,5E+8 (9,1E+7 - 2,3E+8)	1,3E+8 (8,3E+7 - 2,3E+8)	0.153043
Serum paraoxonase/lactonase 3	PON3	2,9E+6 (2,4E+6 - 5,1E+6)	1,6E+6 (1,5E+5 - 5,3E+6)	0.045456
Transthyretin	TTR	1,2E+6 (2,6E+5 - 2,7E+6)	1,1E+6 (3,1E+5 - 2,2E+6)	0.772338
Vesicular integral-membrane protein VIP36	LMAN2	6,8E+5 (6,1E+5 - 7,2E+5)	6,1E+5 (4,3E+5 - 8,0E+5)	0.749119
Vitamin D-binding protein	GC	3,0E+6 (1,4E+6 - 5,9E+6)	3,7E+6 (8,5E+5 - 8,2E+6)	0.775052
Vitronectin	VTN	2,7E+5 (2,2E+5 - 4,4E+5)	2,3E+5 (1,4E+5 - 3,3E+5)	0.081362

MW = Mann Whitney U-test

Supplemental text 3. Correlations found between parameters analysed, not presented in Result section.

Bacterial species

Several correlations between bacterial species was found, such as the amount of *Tanerella forsythia* to *Propionibacterium propionicus* (R-0.65*).

Two-dimensional gel electrophoresis

In LDL/VLDL, the two most basic (highest pI on the gel) isoforms of apoA-I (d and e form) correlated to *Fusobacterium Nucleatum* (r-0.61 and r-0.67, respectively).

In HDL, ApoJ(b) correlated to negatively to plasma glucose levels (r-0.51*), BMI and (0.56*), apoB/apoA-I (r-0.56*) and the glycosylated SAA4(f) form (r-0.64**) and positively to SAA4(a) (r0.59**). There was a negative correlation between the glycated apoA-I and smoking (r-0.58**) and a positive correlation to plasma ALAT (r0.67). ApoJ (b) correlated to *T. Forsythia* and *Treponema Denticola* (r0.54 and r0.57, respectively), and SAA4 f with *T. Denticola* (r-0.59).

nLC-MS/MS

In LDL/VLDL, apoA-I correlated to apoL-1 (r-0.54*), PAF-AH (r-0.75**), *Prevotella intermedia* (r0.59). ApoL-1 correlated to Ig gamma-1 chain C region (r0.83**), PON1 (r0.68**), and plasma apoB/apoA-I (r0.63**). PAF-AH correlated to plasma levels of apoB (r0.53*), apoB/apoA-I (r0.52*) and to *Campylobacter rectus* (r0.56) and *T. denticola* (r0.54). Ig gamma-1 chain C region correlated to *T. Forsythia* (r-0.71) *T. denticola* (r-0.69), *Selenomonas sputigena* (r0.78*) and *P. intermedia* (r-0.58).

In HDL, LCAT levels correlated negatively to pocket depths (r-0.5*) and BOP% (r-0.53*), and positively to apoJ and complement C3 (r0.51*, respectively) and to apoF (0.63*). ApoA-II correlated to alpha-1 antitrypsin (r-61**), apoA-I (r0.61**), apoC-III (r0.68**), SAA1 (r0.68**). Alpha-1-antichymotrypsin correlated to Alpha-2-HS-glycoprotein (r0.69**), apoJ (r-0.67**), PLTP (r-0.64*), PON1 and PON3 (r-0.4* and -0.69**, respectively). ApoC-III correlated to SAA1 (r0.58*). ApoJ correlated to apoF (r0.62**), PLTP (r0.87**), PON1 and 3 (r0.68** and 0.84**), to plasma apoA-I (r0.66**) and apoB/apoA-I (r-0.72**), and to BMI (r-

0.79**). ApoF correlated to complement C3 (r0.66**), PLTP (r0.56*), PON1 and 3 (r0.53* and 0.74**). Complement C3 correlated to PON3 (r0.54*). Ig kappa chain C region correlated negatively to smoking (r-0.50*). PLTP correlated to PON3 (r0.75**), plasma apoA-I (r0.67**) and apoB/apoA-I ratio (r-0.6*). PON3 correlated to apoF (r0.74**), apoM (r0.83**), alpha 1 chymotrypsin (r-0.54*), Alpha-2-HS-glycoprotein (r-0.56*), complement C3 (r0.54*), PON1 (r0.53*), plasma apoA-I (r0.61**) and apoB/apoA-I (r-0.57*). Alpha-1-antichymotrypsin correlated to *Corynebacterium matruchotii* (r0.58), *P. intermedia* (r-0.58) and total bacterial load (r-0.67). ApoA-II correlated to *T. Forsythia* (r-0.64*) and *S. sputigena* (r0.6). ApoC-III correlated to *Propionibacterium propionicus* (r0.71*), *P. gingivalis* (r-0.53) and *T. Forsythia* (r-0.67*). Complement C3 correlated to *P. propionicus* to (r-0.78*). PON3 correlated to *T. Forsythia* (r0.5), *T. denticola* (r0.53) and *P. propionicus* (r-0.5). ApoF correlated to *C. matruchotii* (r0.7*) and *P. intermedia* (r-0.52).

Supplemental Table 4. The concentrations of 71 cytokines, chemokines and growth factors in plasma of patients with periodontitis and healthy controls, analysed by a multiplex immunoassay. * indicates significant (P<0.05) difference between controls and patients. Mann-Whitney U test was used for data that was not normally distributed and Students T-test for normally distributed samples.

	Control (n=9) *Median (min-max) pg/ml*		***Patient (n=10)*** *Median (min-max) pg/ml*	
CTACK	1315.0	(835.7-1632)	1049.0	(638.1-2185.0)
ENA-78	665.0	(32.4-1526)	376.5	(52.2-1191.0) *(P=0.065)*
Eotaxin	88.9	(36.9-295.2)	75.7	(36.3-105.9)
Eotaxin-2	701.3	(546.1-3158)	1133.0	(368.1-4330.0)
Eotaxin-3	43.1	(32.1-77.9)	45.0	(24.1-149.1)
EPO	88.7	(59.6-145)	72.1	(36.9-103.2)
FLT3L	115.8	(77.5-164.6)	119.3	(91.2-172.1)
Fractalkine	10553.0	(7150-11889)	9137.0	(6695.0-11035.0) *****
G-CSF	1.5	(1.3-2.4)	1.9	(0.7-2.6)
GM-CSF	0.0	(0.0-0.3)	0.0	(0.0-0.2)
GRO-α	161.9	(24.7-531.8)	50.3	(33.3-292.2) *(p=0.053)*
I-309	53.6	(45.0-70.3)	56.1	(44.7-68.8)
IFN-α2a	0.1	(0.0-0.1)	0.1	(0.0-0.1)
IFN-γ	7.1	(2.3-53.2)	5.2	(2.8-11.3)
IFN-ß	35.7	(0.0-73.5)	22.7	(11.4-93.7)
IL-10	0.3	(0.2-0.7)	0.2	(0.1-0.4)
IL-12/IL-23p40	10.2	(8.2-21.9)	11.9	(7.3-48.3)
IL-12p70	0.3	(0.1-0.7)	0.2	(0.1-0.7)
IL-13	1.2	(0.4-3.4)	0.9	(0.6-2.3)
IL-15	2.0	(1.7-2.9)	1.9	(1.6-2.7)
IL-16	65.1	(17.4-98.8)	65.2	(39.8-90.0)
IL-17	0.8	(0.0-1.5)	0.6	(0.1-2.7)
IL-17A/F	14.7	(8.2-20.3)	14.4	(7.9-25.3)
IL-17B	2.6	(0.2-4.4)	2.0	(1.3-16.9)
IL-17C	9.7	(0.0-18.7)	5.6	(1.6-17.4)
IL-17D	49.6	(16.8-89.0)	36.4	(26.9-82.1)
IL-17E/IL-25	1.5	(0.8-2.6)	1.6	(0.8-3.1)
IL-17F	196.2	(74.0-329.6)	137.3	(37.0-202.4) *****
IL-18	18.8	(10.921.5)	17.7	(12.0-73.2)
IL-1α	1.4	(0.0-15.3)	1.5	(0.8-19.0)
IL-1RA	174.2	(105.1-340.0)	258.2	(129.9-777.0)
IL-1ß	0.1	(0.0-3.3)	0.1	(0.0-2.4)
IL-2	0.5	(0.3-1.2)	0.4	(0.1-0.7)
IL-21	10.0	(4.3-17.0)	18.0	(6.5-38.6) *****
IL-22	0.8	(0.4-5.5)	1.1	(0.6-2.2)
IL-23	7.0	(3.3-10.7)	8.0	(4.0-14.9)
IL-27	459.5	(356.1-660.8)	353.0	(282.0-719.8)
IL-29	6.0	(3.5-13.3)	10.3	(3.1-35.8)

IL-2Rα	557.7	(279.0-718.3)	385.4	(258.4-833.4)
IL-3	11.3	(4.1-19.0)	7.7	(2.6-16.3)
IL-31	37.3	(21.9-47.3)	32.8	(21.9-52.3)
IL-33	2.6	(1.2-3.5)	1.9	(1.0-4.8)
IL-4	0.1	(0.0-0.1)	0.0	(0.0-0.1)
IL-5	0.6	(0.2-1.4)	0.8	(0.2-2.0)
IL-6	4.7	(3.0-5.5)	4.1	(3.3-6.8)
IL-7	1.7	(1.2-2.8)	1.1	(0.8-2.9) *****
IL-8	6.3	(4.1-23.1)	5.1	(3.4-6.7) *(P=0.085)*
IL-9	12.4	(0.0-19.8)	7.5	(4.8-18.1)
IP-10	338.3	(134.5-1945.0)	149.8	(106.7-3051.0)
I-TAC	40.2	(20.8-96.2)	27.0	(15.5-93.0)
M_CSF	9.9	(7.5-13.6)	10.0	(7.7-15.0)
MCP-1	140.0	(120.3-313.4)	129.9	(104.3-240.8)
MCP-2	27.7	(21.8-34.8)	27.2	(17.8-53.0)
MCP-3	11.0	(9.1-16.5)	11.9	(9.4-13.2)
MCP-4	35.5	(18.8-70.6)	26.7	(19.6-67.2)
MDC	727.5	(511.0-936.2)	882.6	(509.1-1541.0)
MIP	15694.0	(7742.0-111707.0)	17261.0	(10525.0-40774.0)
MIP-1α	26.8	(19.4-36.4)	22.1	(16.6-43.3)
MIP-1ß	40.1	(25.1-69.2)	46.2	(20.8-102.4)
MIP-3α	7.8	(4.9-8.2)	7.4	(5.0-14.4)
MIP-3β	156.9	(128.8-295.0)	196.4	(112.0-1303.0)
MIP-5	4783.0	(3314.0-8586.0)	4859.0	(2345.0-15322.0)
SDF-1α	1827.0	(1253.0-1949.0)	1584.0	(1197.0-2234.0)
TARC	44.0	(20.3-98.1)	38.0	(15.9-101.6)
TNF-α	1.5	(1.3-2.4)	1.4	(1.1-2.5)
TNF-ß	1.5	(1.1-1.9)	1.6	(0.6-2.4)
TPO	38.7	(31.0-46.0)	38.1	(21.1-44.2)
TRAIL	149.7	(90.8-217.6)	166.9	(102.6-282.7)
TSLP	2.1	(1.3-8.0)	1.5	(1.3-4.0)
VEGF-A	21.9	(15.6-47.5)	30.0	(20.0-59.2)
YKL-40	17138.0	(7202.0-257012.0)	17179.0	(9110.0-63247.0)
